# The Autophagy Receptor TAX1BP1 and the Molecular Motor Myosin VI Are Required for Clearance of Salmonella Typhimurium by Autophagy

**DOI:** 10.1371/journal.ppat.1005174

**Published:** 2015-10-09

**Authors:** David A. Tumbarello, Paul T. Manna, Mark Allen, Mark Bycroft, Susan D. Arden, John Kendrick-Jones, Folma Buss

**Affiliations:** 1 Cambridge Institute for Medical Research, University of Cambridge, Cambridge, United Kingdom; 2 MRC Laboratory of Molecular Biology, Cambridge, United Kingdom; Purdue University, UNITED STATES

## Abstract

Autophagy plays a key role during Salmonella infection, by eliminating these pathogens following escape into the cytosol. In this process, selective autophagy receptors, including the myosin VI adaptor proteins optineurin and NDP52, have been shown to recognize cytosolic pathogens. Here, we demonstrate that myosin VI and TAX1BP1 are recruited to ubiquitylated Salmonella and play a key role in xenophagy. The absence of TAX1BP1 causes an accumulation of ubiquitin-positive Salmonella, whereas loss of myosin VI leads to an increase in ubiquitylated and LC3-positive bacteria. Our structural studies demonstrate that the ubiquitin-binding site of TAX1BP1 overlaps with the myosin VI binding site and point mutations in the TAX1BP1 zinc finger domains that affect ubiquitin binding also ablate binding to myosin VI. This mutually exclusive binding and the association of TAX1BP1 with LC3 on the outer limiting membrane of autophagosomes may suggest a molecular mechanism for recruitment of this motor to autophagosomes. The predominant role of TAX1BP1, a paralogue of NDP52, in xenophagy is supported by our evolutionary analysis, which demonstrates that functionally intact NDP52 is missing in Xenopus and mice, whereas TAX1BP1 is expressed in all vertebrates analysed. In summary, this work highlights the importance of TAX1BP1 as a novel autophagy receptor in myosin VI-mediated xenophagy. Our study identifies essential new machinery for the autophagy-dependent clearance of Salmonella typhimurium and suggests modulation of myosin VI motor activity as a potential therapeutic target in cellular immunity.

## Author Summary

One of the most common causes of food poisoning is the pathogen *Salmonella enterica* serovar Typhimurium. This pathogen enters the cells of the body through the intestine and after invasion of these cells it survives and multiplies due to its own ability to evade the immune system, thus causing infection. Understanding how this pathogen evades the natural protective mechanisms present within the cell that normally degrade a foreign body is an important area of current research. Here, we describe a process by which the control of infection is mediated by a cellular self-degradation pathway called autophagy. This pathway requires specific adaptor proteins within the cell that identify the foreign pathogen and target it for degradation. We define the function of a specific adaptor protein required for this process of pathogen recognition and show how this adaptor links to and utilises other cellular machinery, the actin cytoskeleton and associated motor proteins to accomplish this function and restrict pathogen proliferation. Our work thus demonstrates that this specialised autophagy pathway requires the coordination of multiple proteins and we identify novel machinery that is essential to efficiently degrade Salmonella Typhimurium within cells.

## Introduction

Macroautophagy is a catabolic lysosomal degradation pathway utilised by cells to degrade cytosolic aggregated proteins and damaged organelles. In addition, it is a homeostatic process that maintains cell survival and growth under conditions of starvation, as well as acting as a cellular defence mechanism against invading pathogens. Cargo recognition for autophagy-dependent degradation is mediated by selective receptors that identify and facilitate the encapsulation of ubiquitylated substrates through the recruitment of LC3-positive autophagic membranes. This leads to the formation of a double membrane autophagosome, which subsequently matures and fuses with the lysosome, thus leading to degradation and recycling of its cargo.

Autophagy is also a key pathway in innate immunity for the capture and degradation of cytosolic bacteria during infection. Pathogens, such as the gram-negative *Salmonella enterica* serovar typhimurium, enter epithelial cells via actin-rich membrane ruffles and establish residence in a Salmonella-containing vacuole (SCV) for proliferation. Occasionally, the bacteria damages the SCV and escapes into the cytosol, where it rapidly becomes ubiquitylated, which subsequently triggers the recruitment of selective autophagy receptors such as SQSTM1/p62, optineurin as well as NDP52 that target the bacteria for degradation by xenophagy [1–4]. When this pathway is impaired, Salmonella hyper-proliferate in the cytosol compared with their proliferation within the SCV [5, 6].

Interestingly, optineurin and NDP52/CALCOCO2 as well as its paralogue TAX1BP1/CALCOCO3, have been shown to bind directly to the minus-end-directed actin-based motor protein myosin VI, which facilitates autophagosome maturation through endosome delivery. This process requires myosin VI to bind to endosomal adaptors as well as directly binding to the autophagy receptors [7, 8]. Thus TAX1BP1, NDP52 and optineurin appear to serve dual functions as myosin VI cargo adaptor proteins and as autophagy receptors that recruit substrates for degradation, with each containing a LC3 interaction region (LIR) and an ubiquitin-binding domain.

Although optineurin and NDP52 have a well-established function in xenophagy [2, 3, 9], the role of TAX1BP1 in this pathway is unknown. Interestingly, TAX1BP1-deficient mice die prematurely from systemic inflammation in multiple organs, which may be caused by common microbiota that are normally harmless [10]. We therefore set out to compare the requirement of TAX1BP1 and its close paralogue NDP52 in the innate immune response for the clearance of cytosolic bacteria. Here, we report that the conservation of TAX1BP1 protein domain architecture across species, along with its required function, suggest a crucial role for TAX1BP1 in xenophagy.

## Results

### Evolutionary analysis of myosin VI associated adaptors and autophagy-related proteins

**Fig 1:**
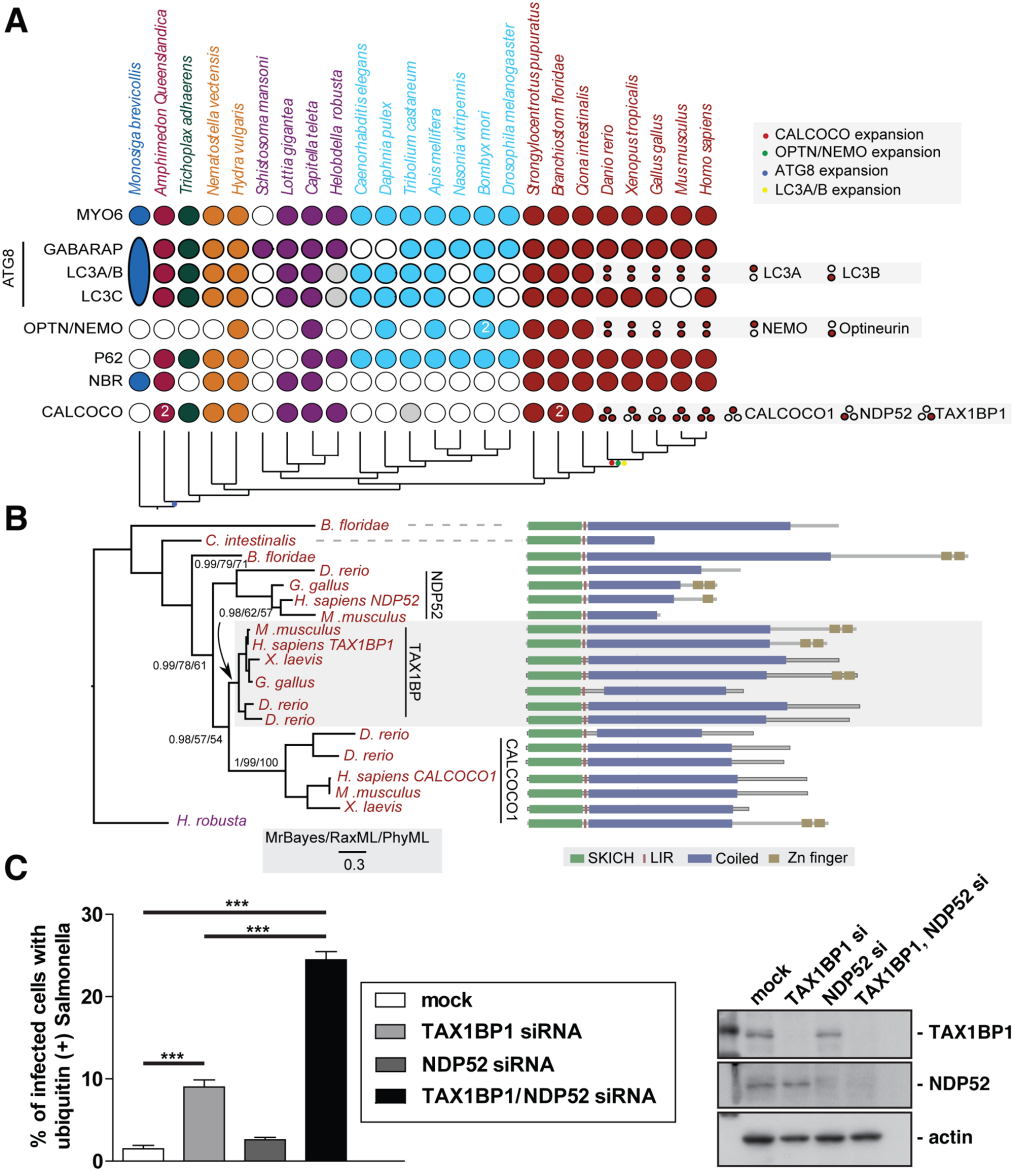
Evolutionary analysis of myosin VI associated adaptors and autophagy-related proteins. (**A**) Coulson plot [30] showing the evolutionary distribution within the holozoa and metazoa of selected myosin VI partners implicated in autophagy. Genes or gene families are represented as rows and taxa are columns. A filled circle indicates the presence of a homolog, empty circles denote instances where no homolog was identified. Grey circles denote identified sequences that could not be reliably placed through phylogenetic reconstruction. Columns are coloured according to the phylogenetic distribution of taxa, indicated with a schematic tree below the table. Unless indicated all homology assignments are supported through phylogenetic reconstruction. Small circles denote paralagous expansion of gene families and where these paralogs have been classified they are stated to the right. Large oval in *M*. *brevicollis* represents the single ATG8 gene within this taxa reflecting the ancestral configuration[31]. Note the expansion of the CALCOCO, OPTN/NEMO and LCA/B gene families occurring at the base of the vertebrates. (**B**) Phylogenetic analysis of the expansion of the CALCOCO gene family. Branch support is shown for major clades. Domain organisation of metazoan CALCOCO gene products corresponding to those in. **(C)** Western blot of lysates harvested from HeLa cells following TAX1BP1 alone, NDP52 alone, or TAX1BP1 and NDP52 double siRNA transfection. HeLa cells transfected with siRNA targeted to TAX1BP1, NDP52, or both together were subjected to an infection with mCherry expressing Salmonella for 8 hours followed by processing for confocal microscopy and quantitation of the % of infected cells with ubiquitin (+) Salmonella. Experiments were performed in triplicate and error bars represent s.d.

To understand the evolutionary relationship between myosin VI and its three binding partners NDP52, TAX1BP1 and optineurin, we carried out a detailed comparative genomic and phylogenetic survey on the relationship between TAX1BP1 and NDP52, as well as myosin VI, other key autophagy receptors and ATG8 homologues (Fig 1A).

Many of the gene families implicated have representatives across metazoans, suggesting an early emergence of the autophagy pathway that would date it to the split from the common ancestor of metazoa and choanoflagellates, although taxonomic coverage in this area is poor and therefore conclusions must remain tentative. We can resolve a second burst of expansion in the autophagy receptors occurring at around the time of the emergence of the vertebrates. During this time, LC3A and LC3B arise from duplication of a common ancestor. An additional expansion gives rise to NEMO and optineurin. Finally the ancestral CALCOCO gene undergoes two apparent duplications leading to the emergence of CALCOCO1, NDP52 and TAX1BP1 (Fig 1A).

The SKICH domain, putative LIR and coiled-coil regions are extremely well conserved features of the CALCOCO gene family. The majority of variation occur at the C-terminal end of the proteins with repeated losses of the zinc finger domains (Fig 1B). It appears, that of the CALCOCO gene family representatives, TAX1BP1 most broadly preserves the ancestral configuration, including the presence of two Zn finger domains at the extreme C-terminus; this feature is seen in the single CALCOCO representative of the leech *H*. *robusta*, a phylogenetically distant taxa whose divergence greatly predates the duplications of the CALCOCO gene family (Fig 1B). Although it should be noted that the NDP52 homologue identified from the chicken, *G*. *gallus*, appears to possess a pair of C-terminal Zn finger motifs (Fig 1B).

Our evolutionary analysis emphasises the importance of TAX1BP1 in vertebrate cells, since functionally intact TAX1BP1 is present in all vertebrate species analysed, whereas NDP52 is lost from Xenopus and only expressed in mice as a truncated form without the C-terminal ubiquitin-binding and Galectin–8 domains.

### Suppression of both TAX1BP1 and NDP52 leads to an accumulation of ubiquitylated Salmonella in the cytosol of infected cells

We next compared whether the absence of TAX1BP1 causes a similar defect in Salmonella clearance as previously described for NDP52 (2). We determined the number of cells containing ubiquitin-positive Salmonella after depletion of TAX1BP1, NDP52 or depletion of both TAX1BP1 and NDP52 by siRNA. Knockdown and mock-transfected HeLa cells (Fig 1C) were infected with Salmonella and 8 hours later the number of cells containing ubiquitin-positive bacteria were quantitated (Fig 1C). Loss of TAX1BP1 causes the accumulation of more cells containing ubiquitin-positive Salmonella compared with NDP52-depleted cells (Fig 1C and S1 Fig). The double KD of TAX1BP1 and NDP52 has a stronger effect than the single KD of either autophagy receptor, indicating that TAX1BP1 and NDP52 may have partially overlapping and redundant functions.

### TAX1BP1 is recruited to ubiquitylated Salmonella

**Fig 2:**
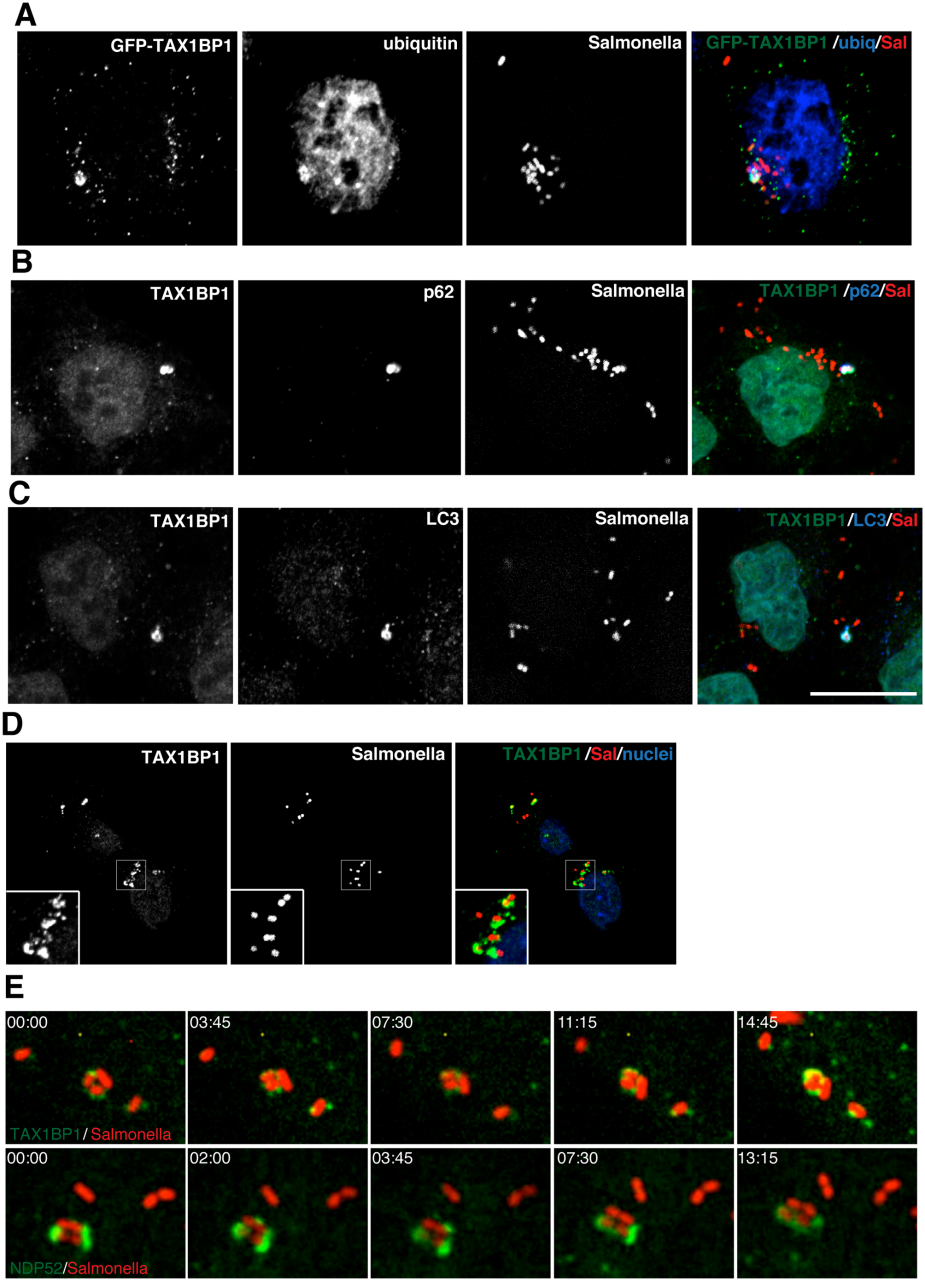
TAX1BP1 localises to Salmonella that have escaped into the cytosol of infected cells. (**A**) HeLa cells transfected with GFP-TAX1BP1 were infected with mCherry expressing Salmonella for 1 hour, processed for confocal immunofluorescence microscopy, and immunostained for ubiquitin. (**B**) HeLa cells infected for 1 hour with mCherry expressing Salmonella were immunostained for TAX1BP1 and p62 or (**C**) TAX1BP1 and LC3. Scale bar, 20 μm. (**D**) Mouse embryonic fibroblasts were infected with mCherry Salmonella for 1 hour and were immunostained for TAX1BP1 and processed for confocal microscopy. Nuclei are labelled with Hoechst (blue) (**E**) Still frames taken from RPE cells expressing GFP-TAX1BP1 or GFP-NDP52 infected with mCherry Salmonella imaged on a spinning disk live cell microscope. Elapsed time in min:sec is displayed.

To further investigate the role of TAX1BP1 during xenophagy, HeLa cells were infected with wild type Salmonella and TAX1BP1 localisation was analysed by immunofluorescence microscopy. GFP-TAX1BP1 (Fig 2A) and also endogenous TAX1BP1 (Fig 2B and 2C) are present on a subset of Salmonella that are also positive for ubiquitin, the autophagy receptor p62 and the autophagy marker LC3 (Fig 2A–2C). We quantified the colocalisation between TAX1BP1 and ubiquitin, which revealed that nearly 100% of ubiquitin-positive Salmonella are also positive for TAX1BP1. Our results are similar to previous findings for NDP52, which demonstrated that at least 75% of ubiquitin-positive Salmonella also contained NDP52 (2). In primary mouse embryonic fibroblasts (MEFs) endogenous TAX1BP1 also shows a prominent recruitment to cytosolic Salmonella (Fig 2D), which is consistent with a dominant role for TAX1BP1 in xenophagy in murine cells, since this species expresses only a truncated form of NDP52 without the C-terminal ubiquitin-binding zinc finger (UBZ).

To visualize how GFP-TAX1BP1 and GFP-NDP52 associate with *S*. typhimurium, we performed time-lapse imaging of mCherry-Salmonella infected HeLa cells. Still images from time-lapse movies illustrate that both proteins appear in discrete punctae on the surface of *S*. typhimurium, which then spread and partially surround the bacteria (Fig 2E; S1 and S2 Movies) indicating that TAX1BP1 and NDP52 are recruited in a similar spatial pattern to cytosolic *S*. typhimurium.

**Fig 3:**
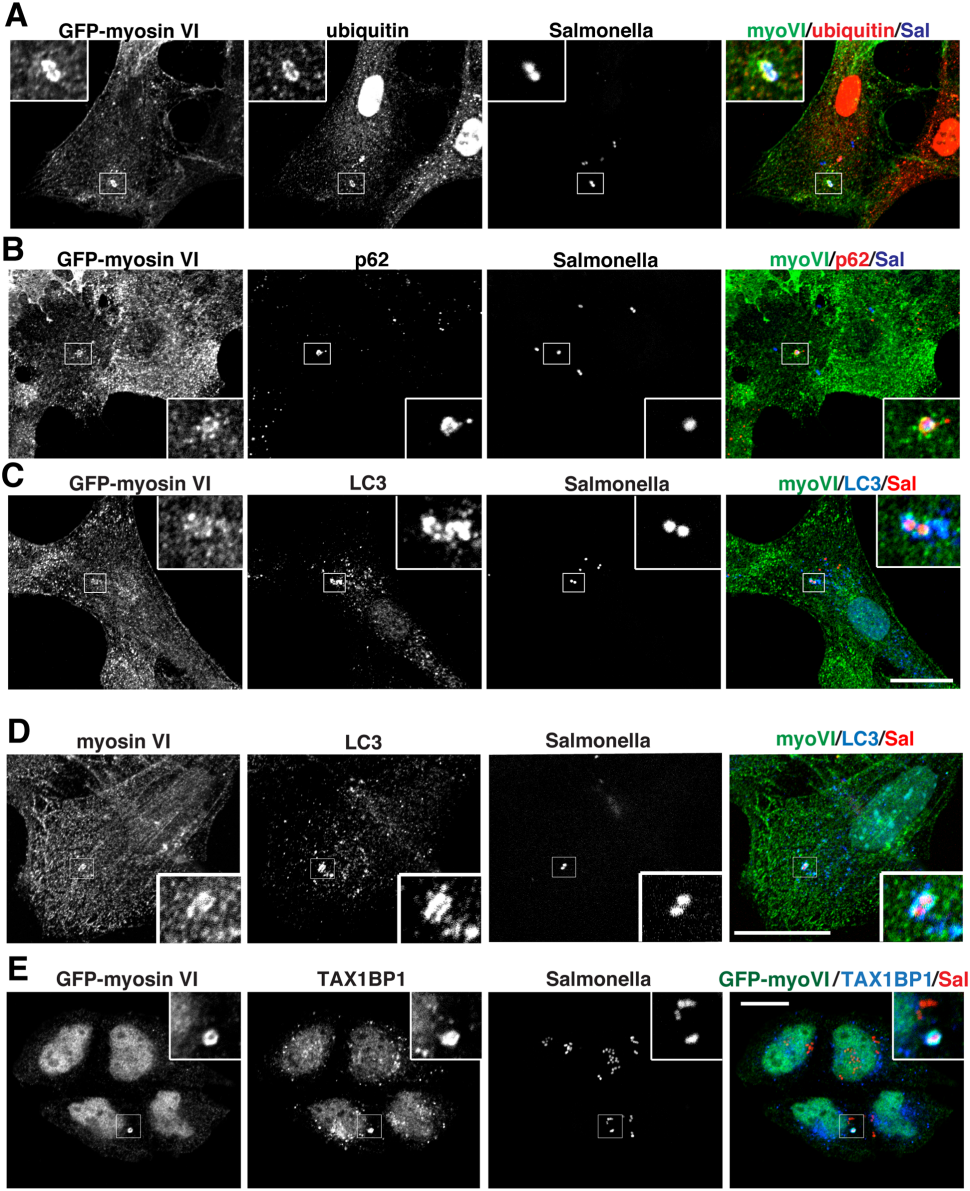
Myosin VI localises to autophagosomes containing Salmonella. RPE cells stably expressing GFP-myosin VI were infected with blue fluorescent protein (BFP) expressing (**A, B**) or mCherry expressing (**C**) Salmonella for 1 hour. Cells were immunostained for ubiquitin (**A**, red), p62 (**B**, red), or LC3 (**C**, blue) and processed for confocal microscopy. (**D**) Mouse embryonic fibroblasts (MEFs) were infected with mCherry-expressing Salmonella and processed for confocal microscopy by immunostaining for myosin VI (green), LC3 (blue) and nuclei were labelled with Hoechst (cyan). (**E**) HeLa cells expressing GFP-myosin VI were infected with mCherry expressing Salmonella (red) for 1 hour, processed for confocal microscopy and immunostained for GFP (green) and TAX1BP1 (blue). Scale bar, 20 μm.

Myosin VI is also recruited to ubiquitylated cytosolic Salmonella that are positive for p62 as well as LC3 (Fig 3A–3D). Furthermore, myosin VI colocalises with autophagy receptors, such as TAX1BP1, on Salmonella present within the cytosol of HeLa cells (Fig 3E).

### TAX1BP1 C-terminal zinc finger domains mediate ubiquitin and myosin VI binding

**Fig 4:**
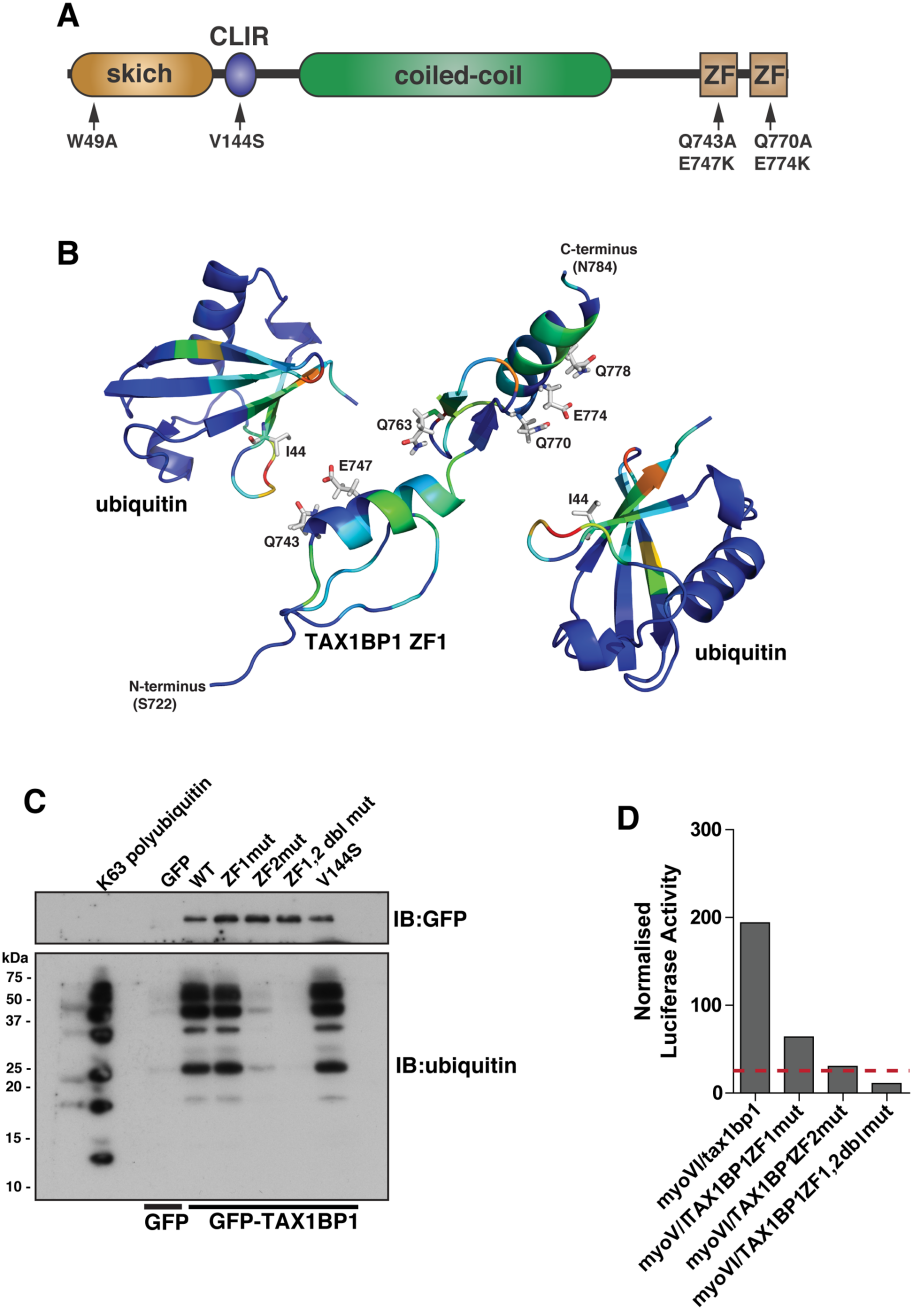
Overlapping binding of TAX1BP1 zinc finger domains with ubiquitin and myosin VI. (**A**) Domain organisation of TAX1BP1 and designated point mutations. LIR, LC3-interaction region; ZF, zinc-finger; SKICH, SKIP carboxyl homology. (**B**) Ribbon presentation of the structures of the TAX1BP1 zinc fingers together with two molecules of ubiquitin. The residues are colored according to the extent of the chemical shift changes observed where blue represents small or no chemical shift changes observed, and red represents the maximum chemical shift changes observed for the backbone amides. The side chains of the residues in TAX1BP1 that when mutated abolish ubiquitin binding are displayed and labelled. (**C**) RPE cells were transfected with GFP empty vector, with GFP-TAX1BP1 wild-type, GFP-TAX1BP1 ZF1 mutant (Q743A/E747K), GFP-TAX1BP1 ZF2 mutant (Q770A/E774K) or the Q743A/E747K/Q770A/E774K double zinc finger mutant of GFP-TAX1BP1 followed by GFP immunoprecipitation and pull-down with K63-linked polyubiquitin (2–7). Western blot analysis was performed and immunoblotting against the indicated proteins. (**D**) Mammalian 2-hybrid assay in CHOK.1 cells with myosin VI tail as bait and TAX1BP1 full-length wild-type and mutants as prey. Results are represented as the normalised luciferase activity against a bait only control.

TAX1BP1 shares a very similar domain organisation with its paralogue NDP52; both proteins contain an amino-terminal SKIP carboxyl homology (SKICH) domain, followed by an atypical LC3-interaction region (LIR) and a central coiled-coil region[11]. At the carboxy-terminus, TAX1BP1 contains two unique ubiquitin-binding zinc fingers (UBZs; compared with one in NDP52), which can bind to K63-linked ubiquitylated proteins such as TRAF6 and ITCH [12, 13] and myosin VI [11] (Fig 4A).

To understand the molecular detail of the TAX1BP1-ubiquitin interaction, we used NMR spectroscopy to perform a structural characterisation of the ZF-ubiquitin complex (Fig 4B). Analysis of the changes produced in the ^1^H/^15^N HSQC spectra of ^15^N labelled TAX1BP1 zinc fingers upon the addition of unlabelled mono-ubiquitin showed alterations in the backbone chemical shifts of residues in both zinc fingers (Fig 4B, S2A Fig and S1 Table). In a reciprocal experiment in which unlabelled TAX1BP1 zinc finger was added to ^15^N labelled mono ubiquitin, the residues that showed changes in backbone chemical shifts were located in the isoleucine 44 patch of ubiquitin (S1 Table). The majority of residues whose backbone chemical shifts alter upon ubiquitin binding in TAX1BP1 are located in the helices of the zinc fingers. The side chain resonances of several glutamine residues in this region of the protein also change upon addition of ubiquitin (Fig 4B and S2C Fig).

To confirm the functional significance of these amino acid residues and to determine any overlap in myosin VI and ubiquitin binding, we mutated two of these residues in each of the TAX1BP1 ZF domains (ZF1—Q743A, E747K; ZF2 —Q770A, E774K). These mutations in the ZF domains do not cause a large conformational change that leads to unfolding of the proteins, as the HSQC spectra of both mutants reveal that they are clearly folded with chemical shifts in similar positions to the wild type proteins (S3 Fig). Mutant GFP-TAX1BP1 containing double point mutations in ZF1 or ZF2 or both was expressed in RPE cells, immunoprecipitated with antibodies to GFP before a pulldown with K63-linked polyubiquitin was performed. Our results clearly show that only ZF2 is required for association with K63-linked ubiquitin chains, whereas mutations in ZF1 have no effect on ubiquitin binding (Fig 4C). To characterise the binding between myosin VI and TAX1BP1, we used the mammalian 2-hybrid assay with the myosin VI cargo-binding tail domain as bait and full-length wild-type and mutant TAX1BP1 as prey. In contrast to ubiquitin binding, the association between myosin VI and TAX1BP1 involves ZF1 as well as ZF2, since point mutations in either ZF decrease myosin VI—TAX1BP1-binding, although to a greater extent following mutations in ZF2 (Fig 4D).

NDP52 and optineurin, along with TAX1BP1, contain a C-terminal zinc finger (ZF) motif but optineurin also has an ubiquitin binding in ABIN and NEMO (UBAN) domain (Fig 4A and S4A and S4B Fig). K63 linked polyubiquitin pull-down assays demonstrate that for optineurin the UBAN and for NDP52 the C-terminal ZF is required for ubiquitin binding (S4C and S4D Fig). Point mutations in these domains that ablate ubiquitin-binding also completely inhibit binding to myosin VI (S4E Fig). Intriguingly, these results demonstrate that in all three autophagy receptors, the ubiquitin-binding site overlaps with the myosin VI binding site thereby confirming our hypothesis on the possible mechanism of myosin VI recruitment to autophagosomes [7, 8].

**Fig 5:**
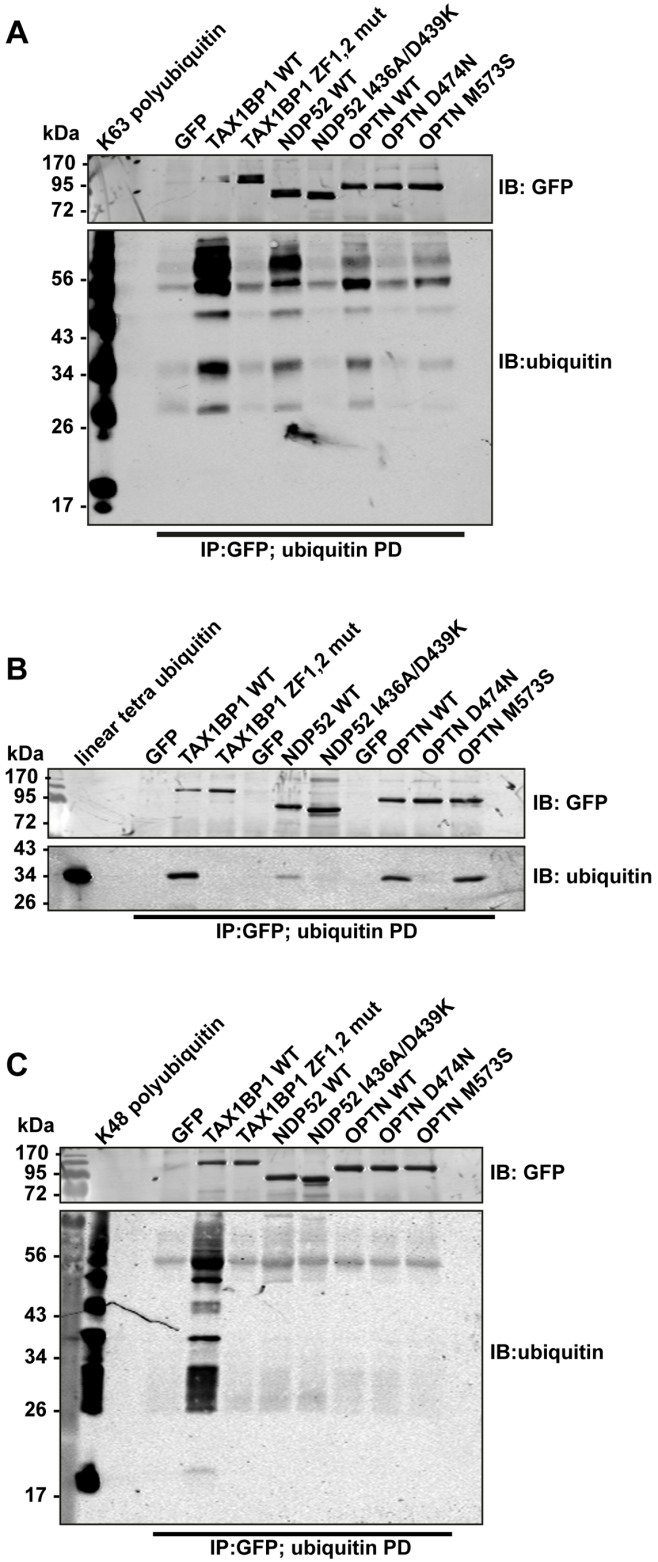
Binding of TAX1BP1, NDP52 and optineurin to different ubiquitin chain types. GFP immunoprecipitation followed by either linear tetra-ubiquitin (**A**), K48-linked polyubiquitin (**B**), or K63-linked polyubiquitin (**C**) pull-downs (PD) from RPE cells transfected with GFP alone, GFP-TAX1BP1, GFP-NDP52, or GFP-optineurin wild-type and mutants. Western blot analysis performed using antibodies specific for ubiquitin and GFP.

We next compared binding of wild-type and ubiquitin binding mutant TAX1BP1, NDP52, and optineurin to different ubiquitin chain types (Fig 5). These experiments show that all 3 autophagy receptors bind K63-linked polyubiquitin (Figs 4C and 5A and S4C and S4D Fig), however, with varying degrees. TAX1BP1 displays the strongest binding to K63 polyubiquitin chains (Fig 5A). In addition, TAX1BP1, NDP52 and optineurin also bind to linear tetra-ubiquitin, although NDP52 binds less well than TAX1BP1 and optineurin (Fig 5B). Interestingly, only TAX1BP1 is capable of interacting with K48-linked polyubiquitin chains (Fig 5C).

**Fig 6:**
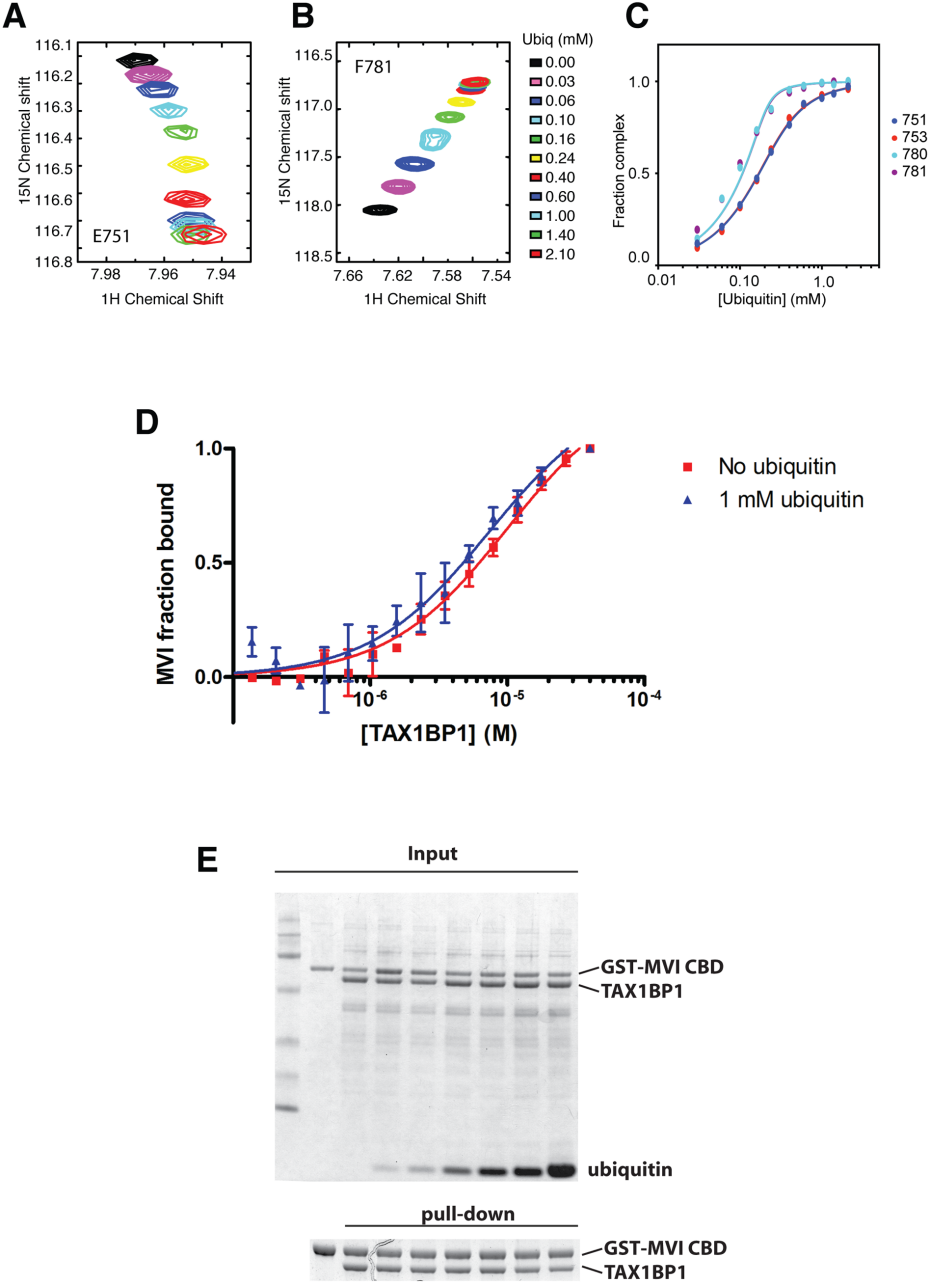
Binding of TAX1BP1 zinc finger domains to ubiquitin and myosin VI. Dependence of the chemical shift changes of resonances of residues in TAX1BP1 as a function of ubiquitin concentration: (**A**) and (**B**) overlay of sections of the ^1^H/^15^N HSQC spectra of ^15^N labeled TAX1BP1 zinc fingers at different concentrations of unlabeled ubiquitin showing the concentration dependence of the peak positions of residues in each of the zinc fingers. (**C**) Graph showing the concentration dependence of these peaks together with two other peaks from each of the zinc fingers. The chemical shift change of the backbone amide group (Δδ_HN−N_) is computed with Δδ_HN−N_ = √ _(δ_H_^2^+ (δ_N_/10)^2^), where δ_H_ and δ_N_ are the changes in ^1^H and ^15^N chemical shift, respectively. If changes in the spectra are observed and the signals are in fast exchange, the dissociation constant (K_D_) is calculated by fitting the NMR titration data to the formula: $\Delta \delta_{\text{HN-N}}=\frac{\delta_{b}-\delta_{f}}{2\left[P_{0}\right]}\left(\left[P_{0}\right]+\left[L_{0}\right]+K_{D}-\sqrt{{\left(\left[P_{0}\right]+\left[L_{0}\right]+K_{D}\right)}^{2}-4\left[P_{0}\right]\left[L_{0}\right]}\right)$ where y is Δδ, δ_b_-δ_f_ is the difference in chemical shift between bound and free state (= Δδ_max_), P_0_ is the total protein concentration and L_0_ is the total ligand concentration. A simple two-state binding model is assumed. Protein concentration is treated as a constant (measured before starting the titration). For our experiments P_0_ was 150 μM. (**D**) The direct interaction of myosin VI CBD with TAX1BP1 was measured as changes in thermophoretic mobility of 75 nM fluorescently labeled myosin VI CBD in the presence of various TAX1BP1 concentrations. The binding affinity of the complex was estimated by a fit to a Hill function, as a *K*_D_ of about 5–10 μM. The experiment was repeated in the presence of 1 mM ubiquitin. Even the presence of a large excess of free ubiquitin does not change the binding affinity between myosin VI and TAX1BP1. Experiments were performed in triplicate (no ubiquitin) or duplicate (1mM ubiquitin) and error bars represent s.d. (**E**) Pull-down assay of myosin VI CBD and TAX1BP1 in the presence of ubiquitin. 15 μM TAX1BP1 was incubated for 30 min with increasing amounts of ubiquitin (0–1 mM) before adding 10 μM GST-myosin VI CBD. After pull-down with Glutathione-Sepharose beads, the amount of TAX1BP1 binding to GST-myosin VI CBD was visualised by SDS-PAGE. Upper gel shows the total input and lower panel the pull-down.

Next, we determined the binding affinities of the two ZF domains on TAX1BP1 for ubiquitin. The NMR data were analysed for the dependence of the chemical shift changes on the amount of ubiquitin added for peaks in each of the TAX1BP1 ZFs. The results in Fig 6A–6C show that the two ZFs bind ubiquitin with differing affinities, 70 μM for N-terminal module (ZF1) and 7 μM for the C-terminal one (ZF2). To compare the binding affinities of the TAX1BP1 ZF domain for myosin VI and for ubiquitin, we performed interaction studies in solution using microscale thermophoresis (MST) [14] and determined an equilibrium dissociation constant (*K*_D_) of around 5 μM for myosin VI—TAX1BP1 binding (Fig 6D). Next, we repeated this experiment and measured the thermophoretic mobility of the labeled myosin VI CBD (75 nM) with increasing concentrations of TAX1BP1 in the presence of 1 mM ubiquitin (Fig 6D). Interestingly, the large excess of mono-ubiquitin was not able to displace TAX1BP1 from myosin VI. These results were confirmed in GST pull-down experiments. Purified GST-myosin VI CBD (10 μM) was incubated with C-terminal TAX1BP1 (aa 291–747) (15 μM) and increasing amounts of ubiquitin (25–1000 μM) (Fig 6E). The GST-myosin VI CBD was able to pull-down similar amounts of TAX1BP1 in the presence of a 100 times excess of ubiquitin, indicating that binding between myosin VI and TAX1BP1 cannot be displaced by a surplus of free ubiquitin (Fig 6E). Taken together our results suggest a dual role for TAX1BP1 in autophagosome formation. The autophagy receptor is first binding with lower affinity, when high amounts of ubiquitin are present, to cytosolic Salmonella, and then preferentially with higher affinity as an autophagy adaptor to myosin VI on the outer limiting membrane of the autophagosome.

### TAX1BP1, NDP52 and optineurin are selective for different LC3 isoforms

**Fig 7:**
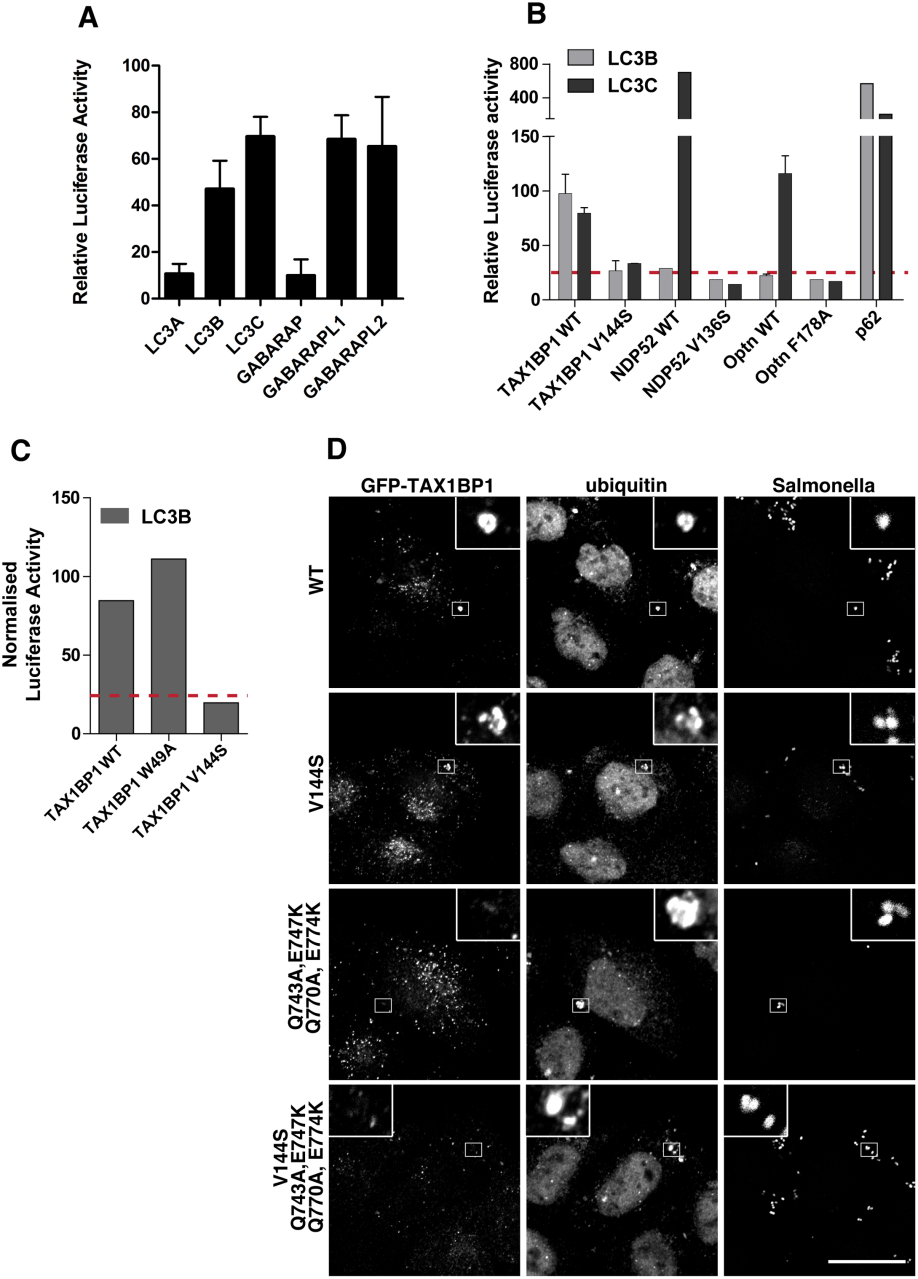
TAX1BP1 interacts with LC3B as well as LC3C and requires its zinc finger domains to localise to ubiquitylated Salmonella. (**A**) Mammalian 2-hybrid assay in CHOK.1 cells with LC3A, B, or C and GABARAP, GABARAPL1 or L2 as bait and TAX1BP1 full-length wild-type as prey. Results are represented as the normalised luciferase activity against a bait only control. **(B)** Mammalian 2-hybrid assay in CHOK.1 cells using LC3B or LC3C as bait and full-length TAX1BP1, NDP52, optineurin wild-type and LIR mutants as prey. Results are represented as the normalised luciferase activity against a bait only control. (C) Mammalian 2-hybrid assay in CHOK.1 cells with LC3B as bait and TAX1BP1 full-length wild-type and mutants as prey. Results are represented as the normalised luciferase activity against a bait only control. (**D**) HeLa cells transfected with GFP-TAX1BP1 wild-type or V144S LIR mutant, Q743A/E747K/Q770A/E774K double zinc finger mutant, and V144S/Q743A/E747K/Q770A/E774K LIR and double zinc finger mutant were infected with mCherry expressing Salmonella for 1 hour prior to saponin extraction and fixation. Cells were immunostained for GFP and ubiquitin and processed for confocal microscopy. Nuclei are labelled with Hoechst (blue). Scale bar, 20 μm.

Several ATG8/LC3 orthologues are expressed in higher eukaryotes and NDP52 interacts specifically via its CLIR with the LC3C isoform, which has an important role in xenophagy [15]. Interestingly, TAX1BP1 is the only known selective autophagy receptor that contains a similar atypical LIR. We therefore first analysed binding of TAX1BP1 to LC3A, B and C as well as GABARAP, GABARAPL1 and L2 using a mammalian 2-hybrid assay. As shown in Fig 7A, TAX1BP1 not only binds to LC3B and C but also to GABARAPL1 and L2. We next compared the binding specificities of NDP52 and TAX1BP1 as well as optineurin to either LC3B or LC3C. As previously reported[15], NDP52 selectively interacts only with LC3C as does optineurin; in contrast to NDP52, optineurin contains a canonical LIR with a bulky aromatic residue in the centre of the binding domain. Surprisingly, TAX1BP1 shows no preference and binds equally to LC3B and LC3C (Fig 7B); this may be due to a methionine residue (rather than an isoleucine present in NDP52) upstream of the TAX1BP1 LVV motif, which is a crucial residue in conferring specificity of NDP52 for LC3C (15). None of the point mutations in TAX1BP1, NDP52 or optineurin that affect LC3 binding lead to a reduction in myosin VI association (S4F Fig).

TAX1BP1, like NDP52, contains two putative LC3-interacting regions, a canonical LIR motif (LIR) in the N-terminal SKICH domain as well as a non-canonical LIR (CLIR) in the linker region connecting the N-terminal SKICH region and the coiled-coil domain. Mutating the crucial aromatic residue in the LIR (W49A) in the SKICH domain does not affect TAX1BP1-binding to LC3B, whereas LC3-binding is completely abolished by a single point mutation in the LVV motif (V144S) of the CLIR (Fig 7C).

### Targeting of TAX1BP1 to cytosolic Salmonella requires ubiquitin binding

Next, we tested which TAX1BP1 protein-binding domains are required for targeting to cytosolic bacteria and mediate its function as a xenophagy receptor. TAX1BP1 is recruited to starvation-induced autophagocytic structures [7, 16] through a non-selective process that can be mediated by either ubiquitin or LC3-binding, but is inhibited by mutation of both together (S5 Fig). In contrast, recruitment of TAX1BP1 to cytosolic Salmonella does not require LC3-binding, but instead depends on ubiquitin-binding (Fig 7D). TAX1BP1 carrying point mutations in the two ZF domains is no longer recruited to ubiquitylated Salmonella. These results identify TAX1BP1 as an autophagy receptor that selects ubiquitylated Salmonella as a substrate for xenophagy via its two zinc finger domains.

### In myosin VI-depleted cells, ubiquitylated Salmonella accumulate inside LC3-positive autophagosomes

**Fig 8:**
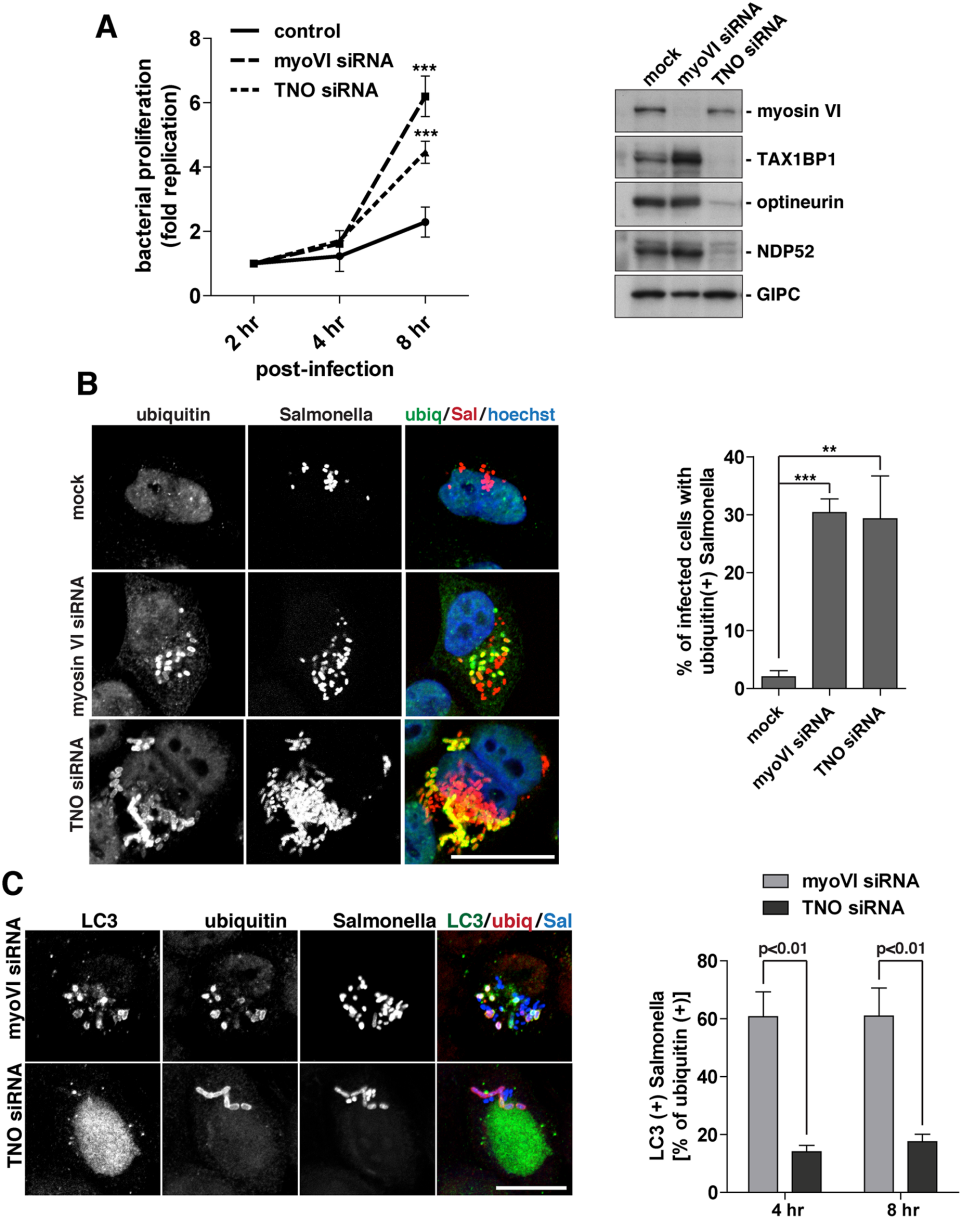
Suppression of myosin VI expression leads to a hyper-proliferation of Salmonella and an accumulation of ubiquitylated Salmonella within LC3-positive autophagosomes. (**A**) HeLa cells following myosin VI siRNA or TAX1BP1, NDP52, OPTN (TNO) siRNA transfection were subjected to Salmonella infection for the indicated time points. Gentamicin protection assays were performed and colonies were counted at each time point. Results are represented as the fold replication from 2 hours, which represents the amount of bacterial proliferation. Results are from >3 independent experiments and error bars represent s.d. Western blot analysis was performed on whole cell lysates and immunoblotting was performed to the indicated proteins. (**B**) HeLa cells mock, myosin VI siRNA, or TNO siRNA treated were infected with mCherry expressing Salmonella for 8 hours, followed by processing for confocal microscopy. Cells were immunostained for ubiquitin (green). Nuclei were labelled with Hoechst (blue). The % of infected cells with ubiquitin (+) Salmonella was quantified. The results depict >3 independent experiments and the error bars represent the s.d. (**C**) HeLa cells transfected with myosin VI or TNO siRNA were infected with BFP-expressing Salmonella for 8 hours. Cells were processed for confocal microscopy and immunostained for LC3 (green) and ubiquitin (red). Scale bar, 20 μm. Quantitation at 4 and 8 hours post-infection, with BFP-expressing Salmonella, of the % of ubiquitin (+) Salmonella which are LC3 (+). Results represent 3 independent experiments and the error bars indicate the s.d.

To compare the function of myosin VI and the autophagy receptors in controlling Salmonella infection, we utilised a Gentamicin protection assay to measure Salmonella proliferation within the cytosol. Internalised Salmonella were harvested from HeLa cells at 2, 4, or 8 hours post-infection in mock and myosin VI or TAX1BP1/NDP52/optineurin (TNO) siRNA depleted cells and the fold replication of Salmonella was measured. We observed a hyper-proliferation of Salmonella in myosin-VI-depleted cells, which is even higher than that seen with the simultaneous depletion of all three autophagy receptor proteins (TNO) (Fig 8A).

Since the loss of myosin VI leads to Salmonella hyper-proliferation, we determined whether this phenotype was due to a defect in targeting these cytosolic bacteria for degradation by autophagy. Thus, at 8 hours post-infection, the number of cells containing ubiquitin-positive Salmonella were quantitated in myosin VI or TNO depleted cells. Our results indicate a very similar, abundant accumulation of cells with ubiquitin-positive Salmonella in cells lacking myosin VI or the three autophagy receptors (Fig 8B), indicating that the xenophagy pathway is impaired.

Previous studies have demonstrated that Salmonella with a mutation in the sifA protein, the pathogenicity island 2 type-III effector, has a defect in maintenance of the Salmonella-containing vacuole (SCV), which leads to an increased tendency to escape into the cytosol and hyper-proliferate in epithelial cells [17, 18]. Using the unique characteristics of the sifA mutant Salmonella we determined whether loss of myosin VI expression potentiates this hyper-proliferation phenotype by inhibiting xenophagy. HeLa cells depleted of myosin VI protein expression by siRNA transfection exhibited an accumulation of cells with ubiquitin positive (+) ΔsifA Salmonella at 1, 4, and 8 hours post-infection (S6A and S6B Fig). These Salmonella not only become ubiquitylated (S6 and S7B Figs) but also colocalise with LC3 and p62 (S6 Fig). Interestingly, the mutant Salmonella appear as fused tubes likely due to a septation defect following replication (S6A Fig). This phenotype was not only observed in HeLa cells, but also in MEFs infected with a sifA mutant strain of Salmonella, which may indicate a particular defect in the bacterial septation machinery for those Salmonella trapped within an autophagosome, potentially due to changes in pH or impacts by other host factors.

In order to verify that this phenotype was not due to off-target effects as a result of siRNA transfection, we performed rescue experiments using a stable HeLa cell line that expresses a siRNA resistant form of myosin VI. The ectopic expression of siRNA resistant GFP-myosin VI is able to rescue the phenotype exhibited by depletion of endogenous myosin VI, resulting in a decrease in the accumulation of cells with ubiquitin (+) Salmonella (S6C, S6D and S6E Fig).

In addition to these rescue experiments, we also analysed Salmonella clearance in embryonic fibroblasts isolated from Snell’s waltzer myosin VI KO mice. Primary wild-type and myosin VI KO fibroblasts were harvested and infected with Salmonella for a period of 2, 4, and 8 hours and the rate of proliferation was measured using a Gentamicin protection assay. We observed a significant hyper-proliferation of Salmonella in myosin VI KO cells compared to wild-type mouse fibroblasts (S7A Fig). When we infected the wild-type and myosin VI KO cells with ΔsifA Salmonella for a period of 1, 4, or 8 hours, we observed an increase in the signal for ubiquitin-positive Salmonella in myosin VI KO fibroblasts visualised by confocal microscopy and quantitated by automated high-throughput microscopy, which again, characteristically exhibit a tubular phenotype due to a likely septation defect following Salmonella replication (S7B and S7C Fig).

In order to determine whether these ubiquitin-positive Salmonella were present within autophagosomes or accumulated in the cytosol, we next co-stained with the autophagosome specific protein LC3. Loss of the autophagy receptors, TAX1BP1, NDP52 and optineurin, leads to an accumulation of ubiquitin-positive Salmonella, which are unable to be degraded, due to their inability to recruit LC3-positive autophagosomal membranes (Fig 8C) (2, 3). In contrast, loss of myosin VI results in an accumulation of Salmonella positive for both ubiquitin and LC3 (Fig 8C). These results suggest that myosin VI depleted cells arrest at a later stage along the autophagy pathway, resulting in the accumulation of Salmonella inside LC3-positive autophagosomes (Fig 8C).

In summary, these results show a clear requirement for myosin VI in autophagy-dependent clearance of cytosolic bacteria. In contrast to loss of TAX1BP1, NDP52 and optineurin, which causes an accumulation of cytosolic ubiquitylated Salmonella, depletion of myosin VI dramatically increases the number of bacteria inside autophagosomes, indicating a defect later in autophagosome degradation.

## Discussion

Selective autophagy is an important cellular mechanism that limits the spread of Salmonella infection, since defects in this pathway lead to the hyper-proliferation of Salmonella within the cytosol of infected cells [1]. Several autophagy receptors are required for clearance of cytosolic Salmonella, including NDP52 and optineurin, and to some extent also p62 [3, 4, 19]. But it has not been clear whether these autophagy receptors have unique functions in this process or overlapping redundant roles.

Here, we provide evidence for the requirement of TAX1BP1 as a novel receptor protein in xenophagy and highlight both redundant and non-redundant roles for autophagy receptors. In addition our results clearly demonstrate a crucial requirement for myosin VI at a late step in xenophagy. We illustrate that TAX1BP1 is recruited to ubiquitylated Salmonella and TAX1BP1 binding to ubiquitin and myosin VI requires its C-terminal zinc finger domain. Moreover, TAX1BP1, NDP52 and optineurin have overlapping ubiquitin and myosin VI binding regions, but are selective for different LC3 isoforms. TAX1BP1 and NDP52 both possess a non-canonical LIR motif (CLIR), which is missing the key aromatic residue found in the central position of the canonical LIR [20]. Previous studies have shown that in NDP52 the central LVV motif within the CLIR is crucial for binding to LC3C [15]. Our results confirm that NDP52 selectively binds to LC3C and not to LC3B; a preference that is shared with optineurin, the second autophagy receptor that is important in xenophagy (Fig 7B). Surprisingly, TAX1BP1 does not display this selective binding to only LC3C, although it contains the same non-canonical LIR motif, LVV. However, TAX1BP1 contains a methionine instead of an isoleucine preceding the LVV sequence and it has been shown that mutating the isoleucine to a tryptophan in NDP52 abolishes its selective binding to LC3C [15] suggesting that this amino acid residue is critical to confer specificity. The ability of TAX1BP1 to bind not only to LC3B but also to LC3C, GABARAPL1 and L2 may explain why loss of TAX1BP1 causes a stronger defect in xenophagy compared to loss of NDP52 in our experiments (Fig 1C). Furthermore, not only NDP52 but also optineurin selectively binds to LC3C, and may therefore also play a role in recruitment of LC3C to ubiquitin-coated Salmonella (Fig 7B). Thus, these data suggest a non-redundant mechanism to recruit a wider spectrum of membranes containing other LC3 orthologues such as LC3B or GABARAPs, enabling the phagophore to grow into an autophagosome to capture large cytosolic bacteria.

Our previous work demonstrated that myosin VI mediates the delivery of endocytic membranes to autophagosomes, a process that is required for autophagosome maturation and its fusion with the lysosome [7]. Here, we now show that the ubiquitin binding site in all three adaptor molecules overlaps with the myosin VI binding site and single point mutations that affect ubiquitin binding also affect myosin VI association (Fig 4 and S4 Fig). Interestingly, our binding studies show that the interaction of TAX1BP1 with myosin VI is of higher affinity than binding of TAX1BP1 to ubiquitin, which suggests a possible mechanism for recruitment of myosin VI-containing endosomes to the surface of autophagosomes [8]. In this model TAX1BP1 can function as an autophagy adaptor binding LC3 on the outer limiting membrane of the autophagosome. In this location the two TAX1BP1 ZF-domains no longer bind to ubiquitylated Salmonella, but instead recruit myosin VI associated either with endosomes or from a cytosolic pool [8].

Our results show that loss of myosin VI causes a strong inhibition of Salmonella clearance; a similar level can only be achieved for the autophagy receptors/adaptors if the expression of TAX1BP1, NDP52 as well as optineurin is reduced simultaneously in a triple knockdown experiment by siRNA (Fig 8A). Interestingly, loss of myosin VI function specifically leads to an accumulation of ubiquitylated Salmonella inside LC3-positive autophagosomes, rather than ubiquitin-positive Salmonella that accumulate in the cytosol following autophagy receptor depletion, thus implicating myosin VI as a critical factor during the later maturation stage of the autophagosome. This is consistent with our previous analysis of basal autophagy and suggests that at least some of the machinery enabling autophagosome degradation is conserved across different autophagy pathways [7]. One open question is the mechanistic role of myosin VI and how it facilitates autophagy progression and subsequent fusion with the lysosome. Our data supports a model whereby myosin VI mediates sorting and delivery of endosomal membrane-associated cargo to autophagosomes. In this process, myosin VI may bring endosomes into close contact with autophagosomes and tether them in the surrounding actin cytoskeleton by binding to its endosomal cargo adaptor TOM1 and to the autophagy adaptors TAX1BP1, NDP52 or optineurin through two distinct sites in the cargo binding tail domain.

It is important to understand why so many different autophagy receptors/adaptors, (optineurin, p62, NDP52 and now TAX1BP1) are recruited to ubiquitylated cytoplasmic Salmonella. Several studies have demonstrated that depletion of single autophagy receptors reduces the efficiency of antibacterial autophagy. Interestingly, simultaneous depletion of NDP52 and p62 does not give an additive increase in impairment of Salmonella clearance as compared with individual siRNA depletion, indicating that NDP52 and p62 fulfill cooperative, non-redundant functions [21]. Interestingly, the simultaneous loss of TAX1BP1 and NDP52 gives rise to a stronger defect than observed in cells depleted of either TAX1BP1 or NDP52 alone, suggesting that these two receptors may perform partially redundant overlapping functions in xenophagy (Fig 1C). However, in all our experiments using wild-type or mutant Salmonella loss of TAX1BP1 causes a significant higher accumulation of ubiquitylated bacteria as compared to loss of NDP52, suggesting that expression of TAX1BP1 can partially compensate for the loss of NDP52 but not vice versa (Fig 1C). In mice, a truncated form of NDP52 is expressed that lacks the ubiquitin, Galectin–8, and myosin VI binding domain, and therefore we predict would be unable to compensate for the loss of TAX1BP1. Thus, this may account for the TAX1BP1 KO mice suffering from commensal microbiota infection, which are normally controlled by autophagy [10].

In order to gain further insight into the relationship and functional overlap between TAX1BP1 and NDP52, we undertook an evolutionary investigation. Our phylogenetic analysis reveals that the three paralogues CALCOCO1, TAX1BP1 and NDP52 arose as the result of a gene duplication event in early vertebrates. With the available phylogenetic resolution these expansions appear to have occurred in an evolutionarily short timeframe and it is interesting to speculate that this signals the emergence of increased selectivity or specificity within the autophagic pathway. Indeed, NDP52 has evolved to display more selective substrate specificity with only binding to LC3C as compared to TAX1BP1.

Importantly, whereas TAX1BP1 is well-conserved and found in all vertebrates analysed, NDP52 is completely lost from *Xenopus* and a truncated form of NDP52 exists in mice. The fact that TAX1BP1, in contrast to NDP52, is retained in all vertebrates highlights its importance and implies that TAX1BP1 fulfils essential cellular roles that cannot be compensated for by other autophagy receptors including its close paralogue NDP52. Intriguingly, mutant forms of NDP52 adapt to serve different cellular roles; for example, in mice, the truncated form of NDP52 binds to hyper-phosphorylated tau via its SKICH domain and thereby targets pathological tau for autophagy-mediated degradation by a novel mechanism [22]. Thus, the selectivity of autophagy receptors for cargo and their spatial and temporal function during autophagosome biogenesis is an important avenue for further investigation. Interestingly, new data indicates that NDP52 is not only required during autophagosome formation, but also may play a role in myosin VI-dependent autophagosome maturation [9]. It is clear that there is partial redundancy during autophagy to ensure proper function of a critical cellular pathway, but crucial non-redundant functions do exist that likely confer specificity together with providing unknown regulatory functions that have yet to be fully explored.

## Materials and Methods

### Reagents and antibodies

The following commercial antibodies were used in this study: monoclonal antibodies to p62 (BD Biosciences), LC3 (MBL), GFP (Abcam), ubiquitin (clone FK2; Enzo); polyclonal antibodies to Tom1/Tom1L2 (Abcam), GIPC (Santa Cruz), actin (Sigma), Salmonella (Abcam), and GFP (Life Technologies). Biotin-conjugated anti-ubiquitin antibody, clone P4D1, was purchased from eBiosciences. Rabbit polyclonal antibodies against myosin VI, TAX1BP1, NDP52, and optineurin were produced as previously described [11, 23, 24].

Human recombinant linear tetra-ubiquitin (Ub4), K63-linked polyubiquitin chains (2–7), and K48-linked polyubiquitin chains (2–7) were purchased from BostonBiochem (R&D Systems Europe Ltd.).

### Plasmids

Site-directed mutagenesis was performed on TAX1BP1 cDNA to create W49A (LIR mutant), V144S (CLIR mutant), Q743A/E747K (ZF1 mutant), Q770A/E774K (ZF2 mutant), Q743A/E747K/ Q770A/E774K (ZF 1 and 2 double mutant) and all were subcloned into pEGFPc vector. All mutagenesis was confirmed by sequencing. For the mammalian 2-hybrid assay, cDNA was subcloned into pVP16 prey vector. pEGFPc2 NDP52 V136S (CLIR mutant) and I436A/D439K (ZF mutant) were produced by site-directed mutagenesis followed by subcloning into pVP16 prey vector. pEGFPc2 optineurin F178A (LIR mutant), D474N (UBAN mutant), D478G (UBAN mutant), V572S (ZF mutant), and M573S (ZF mutant) were produced by site-directed mutagenesis followed by subcloning into pVP16 prey vector. The LC3A, B, C and GABARAP, L1, L2 cDNAs were a kind gift from F. Randow (MRC LMB, Cambridge, UK) and were subcloned into the pM bait vector.

### Cell culture

RPE cells were cultured in DMEM:Ham’s F–12 (50:50), 10% FBS, 2 mM Glutamine, 100 U/ml penicillin and 100 μg/ml streptomycin. HeLa cells were cultured in RPMI–1640, 10% FBS, 2 mM Glutamine, 100 U/ml penicillin and 100 μg/ml streptomycin. All cDNA cell transfections were performed with Fugene6 transfection reagent (Promega) according to the manufacturer’s instructions. For siRNA experiments, ON-TARGETplus SMARTpool or individual siRNA (ThermoScientific) oligonucleotides were transfected into cells with oligofectamine according to the manufacturer’s instructions (Invitrogen).

Mouse embryonic fibroblasts were harvested from wild-type and Snell’s Waltzer mice at E13-E15. Briefly, embryos were dissected from euthanized pregnant females and heads and internal organs were discarded. The rest of tissue was minced and incubated at 37°C for 30 minutes in trypsin and DNAse. Cells were washed once in complete DMEM containing 10% FBS, 2 mM Glutamine, 100 U/ml penicillin and 100 μg/ml streptomycin and triturated with a glass Pasteur pipette. Cells were resuspended in complete DMEM and plated onto tissue culture treated dishes. The following day (passage 1), cells were subsequently used for experiments or frozen as early passage stocks.

### Mammalian two-hybrid assay

The mammalian 2-hybrid assay was performed as previously described [25]. Briefly, CHO cells, cultured in Ham’s F–12, 10% FBS, 2 mM Glutamine, 100 U/ml penicillin and 100 μg/ml streptomycin, were transfected using Fugene6 reagent (Promega) along with the respective bait and prey plasmids, pM and pVP16, and the luciferase reporter plasmids pG5luc and pRL-CMV. Following 48 hour incubation, the cells were processed for luciferase reporter activity using the Dual Luciferase Reporter Assay (Promega). The relative luciferase activity was quantitated and at least 3 independent experiments were performed.

### Immunofluorescence microscopy

Cells were plated on glass coverslips and fixed in 4% formaldehyde followed by permeabilisation in 0.02% Triton X–100 in PBS. Alternatively, to reduce cytoplasmic background, cells were extracted in 0.02% saponin in PBS for 20 seconds prior to fixation. Fixed cells were blocked in 1% BSA in PBS, prior to incubation with primary antibodies at room temperature for 2 hours. Alexa fluor 488, 568 or 647 conjugated secondary antibodies (Invitrogen) were used for detection. Images were captured on a Zeiss LSM710 confocal microscope with Zeiss ZEN software. Live cell video microscopy was acquired on a Zeiss AxioObserver Z1 inverted microscope with a Plan-Apochromot 63X/1.40 oil DIC objective and an AxioCam MR3 equipped with a spinning disk module using Zeiss ZEN software. For live cell studies, images were captured every 10 seconds for a period of 20 minutes. Images were processed with Adobe Photoshop CS4 and assembled in Adobe Illustrator CS4.

Manual quantitation of the % of Salmonella infected cells with ubiquitin-positive Salmonella was performed in >200 cells from at least 3 independent experiments. For quantitation of the % of ubiquitin-positive Salmonella containing LC3, >150 bacteria were counted from multiple cells from 3 independent experiments. In addition, automated quantitation of ΔsifA Salmonella infected mouse embryonic fibroblasts was performed using an Arrayscan VTi HCS microscope (Cellomics) using the Spot Detective.V4 algorithm application. Results were normalised per cell and represent >1000 cells from 3 independent experiments.

### Immunoprecipitation and western blot

For ubiquitin pull-down assays, RPE cells were first transiently transfected using PEI reagent with GFP-tagged TAX1BP1, NDP52, or optineurin constructs. After 24 hours, cell lysates were harvested into immunoprecipitation buffer (50 mM Tris pH 7.4, 150 mM NaCl, 0.2% Triton X–100, 1 mM EDTA, and protease inhibitor cocktail), subjected to GFP immunoprecipitation by incubation with anti-GFP antibody for 2 hours at 4°C followed by addition of 25 μl of Protein A-sepharose (50 mg/ml) and incubation for an additional 1 hour at 4°C. Beads were washed 4 times each in 1 ml of IP buffer, before addition of 0.5 ml of IP buffer and incubation with 1 μg of recombinant linear tetra-ubiquitin, K48 polyubiquitin (2–7), or K63 polyubiquitin (2–7) for 1 hour at 4°C. Beads were subsequently washed 4 times each in 1 ml of IP buffer, before addition of 2x SDS-sample buffer and processing for Western blot analysis. For detection of ubiquitin chains, PVDF membrane was probed with a biotin-ubiquitin antibody conjugate before addition of a HRP-streptavidin secondary antibody.

Whole cell lysates were harvested in 2x SDS-Sample buffer and boiled 5 minutes prior to loading alongside the Precision Plus Dual colour protein standard (BioRad) on 10% SDS-PAGE. Wet transfer of protein to Immobilon-P PVDF (Millipore) was performed, and membranes were blocked with Tris-buffered saline (TBS) containing 5% non-fat dry milk or 3% BSA. Membranes were immunoblotted with the appropriate primary antibody and horseradish peroxidase conjugated secondary antibodies (Sigma-Aldrich) followed by incubation with ECL detection reagent (GE Healthcare Life Sciences, Buckinghamshire, UK).

### Protein purification and GST-pull down assays

TAX1BP1 (aa 291–747) was expressed in pRSET-A vector and purified as described [11] and the GST-myosin-VI-CBD (aa 1030–1277) in pGEX-4T1 vector [26]. TAX1BP1 (15 μM) was incubated 30 min on ice with GST-myosin VI–CBD (aa 1030–1277) (10 μM) in 25 μl wash buffer (100 mM NaCl, 20 mM HEPES pH 7.4, 1 mM MgCl_2_, 5 mM azide, protease inhibitor, 0.5 mM β-mercaptoethanol, 1% glycerol) either without or in the presence of increasing amounts of mono-ubiquitin (up to 1 mM). To perform the pull-down, 15 μl washed GST-Sepharose beads were added and incubated a further 30 min at 4°C. The beads were pelleted and washed 3x with 500 μl of wash buffer before extracting with 2% SDS in the same buffer. After adding SDS-sample buffer aliquots were run on 10% Nupage acrylamide gels with either MOPS-SDS or MES-SDS running buffer (Novex Life Technologies).

### Microscale thermophoresis (MST)

The microscale thermophoresis (MST) method has been described in detail elsewhere[27]. GST-myosin VI–CBD was labeled using the Monolith NT Protein Labeling Kit NHS RED (NanoTemper Technologies GmbH) according to the supplied protocol. The concentration of labeled GST-myosin VI–CBD was kept constant at 75 nM. The concentration of purified TAX1BP1 was serially diluted 2:1 with buffer from μM down to 91 nM. The experiment was repeated in the presence of a fixed concentration of ubiquitin (1 mM). MST measurements were performed using a NanoTemperMonolithNT.115 instrument (NanoTemper Technologies GmbH) at 60% LED and 80% MST power. The equilibrium dissociation constants (*K*_D_) were calculated by fitting the resulting binding curve according to the law of mass action.

### NMR

Wild-type TAX1BP1 zinc finger domain samples were prepared for NMR spectroscopy experiments in 1.0 mM in 90% H_2_O and 10% D_2_O in PBS containing 10 mM β-mercaptoethanol. All spectra were acquired with either a Bruker Advance 700 or a DRX600 spectrometer at 20°C, and referenced relative to external sodium 2,2-dimethyl-2-silapentane-5-sulfonate (DSS) for proton and carbon signals, or liquid ammonium for nitrogen. Assignments were obtained by standard NMR methods using ^13^C/^15^N-labeled and ^15^N-labeled samples. To map the binding interface between the TAX1BP1 zinc fingers and ubiquitin, we collected a series of ^1^H-^15^N HSQC spectra of the ^15^N-labelled TAX1BP1 zinc finger domain in the presence of increasing molar ratios of unlabelled ubiquitin; a reciprocal titration was carried out with the unlabelled TAX1BP1 zinc finger domain added to ^15^N-labelled ubiquitin. Dissociation constants were obtained by fitting the concentration dependence of the normalized chemical shift changes to a single site-binding model. The coordinates and the NMR restraints have been deposited in the PDB with the codes PDB: 5aas and r5aasmr, respectively.

### Salmonella culture, infection and Gentamicin protection assay

*Salmonella enterica* serotype typhimurium (strain 12023) were obtained from F. Randow (MRC LMB, Cambridge, UK) and sifA mutant Salmonella were obtained from D. Humphreys (Department of Pathology, University of Cambridge, UK). Each were grown in Luria Broth (LB) containing the appropriate antibiotic at 37°C. Overnight cultures of wild-type Salmonella typhimurium expressing mCherry cDNA or sifA mutant Salmonella were diluted 1:33 into Luria Broth (LB) containing ampicillin and cultured for 3 hours at 37°C on shaker. Cells to be infected were serum-starved in serum-free culture media containing 0.1% BSA for 3 hours at 37°C. For infection, Salmonella were diluted 1:100 into serum-free media and added to cells for 15 minutes. After which, the cells were washed and fed with complete growth media containing 100 μg/ml Gentamicin. After 2 hours, cells were cultured in complete media with 20 μg/ml Gentamicin. For Salmonella proliferation assays, cells were washed 2x in DPBS and lysed in PBS containing 0.1% Triton-X 100. Samples were serially diluted, plated onto duplicate LB agar plates, and incubated overnight at 37°C. Bacterial colonies were counted from duplicate plates from at least 3 independent experiments and represented as the fold increase from the 2-hour time point. For immunofluorescence microscopy experiments, infected cells were fixed in formaldehyde at the indicated times post-Salmonella infection.

### Comparative genomics and phylogenetics

Homology searches were performed with BLAST using human sequences as queries. Retrieved sequences were verified by reciprocal BLAST against the human database. Where no orthologs were identified, further searches were carried out using relevant sequences from closely related taxa where available. Retrieved sequences were parsed through the InterPro database (v.48) (European Molecular Biology Laboratory—European Bioinformatics institute: http://www.ebi.ac.uk/interpro) to identify conserved domains. A candidate sequence was considered a potential ortholog based upon the presence of conserved domains, high scoring reciprocal BLAST against the initial query sequence, and inspection of protein sequence alignments (MAFFT) for conserved regions. All potentially orthologous sequences were then subjected to phylogenetic analysis. For phylogenetic reconstruction, protein sequences were aligned using MergeAlign (http://mergealign.appspot.com/) [28] or MAFFT [29] and edited manually to remove gaps and poorly conserved regions. Phylogenetic trees were reconstructed using Bayesian (MrBayes) and maximum likelihood (RaxML, PhyML) approaches. PhyML was run *via* the South of France Bioinformatics Platform web server (www.atgc-montpellier.fr/phyml/). RaxML and MrBayes were run via the Cyberinfrastructure for Phylogenetic Research (CIPRES) Science Gateway web server (www.phylo.org). MrBayes version 3.1.2 analyses were run for at least 1 x 10^6^ generations and until convergence, removing all trees before plateau as burn-in.

The accession numbers for the sequences are shown in S2 Table. In addition all sequences, alignments and trees used in this analysis are available from the authors on request.

### Ethics statement

The Snell’s waltzer mice were bred and housed under pathogen-free conditions in the animal facility at Cambridge University. Experimentation involving animals was carried out under a UK Home Office Project Licence granted to Dr Folma Buss (PPL 80/2372) and was approved by the UK Home Office and the University of Cambridge Animal Welfare and Ethical Review Committee. The work has been carried out in accordance to the UK Animals (Scientific Procedures) Act 1986 and follows the Laboratory Animal Science Association (LASA) Guidelines.

## Supporting Information

S1 Fig Suppression of both TAX1BP1 and NDP52 leads to an accumulation of ubiquitylated Salmonella in the cytosol of infected cells. HeLa cells transfected with siRNA targeted to TAX1BP1, NDP52, or both together were subjected to an infection with mCherry expressing Salmonella for 8 hours followed by processing for confocal microscopy. Immunostaining was performed against ubiquitin. Nuclei were labelled with Hoechst (blue). Scale bar, 20 μm.
(TIF)

S2 Fig NMR analysis of TAX1BP1 ubiquitin interaction. (**A**) Normalized cumulative chemical shift for the backbone amide groups of ^15^N-labeled TAX1BP1 zinc finger domains upon addition of unlabeled ubiquitin, (**B**) reciprocal cumulative chemical shift changes upon titration of ^15^N-labeled ubiquitin with unlabeled TAX1BP1 zinc finger domains, (**C**) section of the the^1^H/^15^N HSQC spectra of ^15^N labeled TAX1BP1 zinc fingers showing the resonances of the side-chain NH groups of glutamine residues in the presence and absence of unlabeled ubiquitin. The peaks that change chemical shift or decrease in intensity are labeled.
(TIF)

S3 Fig NMR analysis of mutant TAX1BP1. HSQC spectra of the ZF1 (**A**) and ZF2 (**B**) mutants of TAXBP1 (black) overlayed with the HSQC spectrum of wild-type TAXBP1 (red). Both of the mutant proteins are clearly folded with chemical shift in similar positions to those in the wild-type domain.
(TIF)

S4 Fig NDP52 and Optineurin have overlapping ubiquitin and myosin VI binding regions, whereas mutations in the LIR domain have no effect on myosin VI binding. (**A**) Domain organisation of optineurin with designated point mutations. LIR, LC3-interaction region; UBAN, ubiquitin-binding domain in ABINs (A20-binding inhibitors of NF-κB) and NEMO; ZF, zinc-finger. (**B**) Domain organisation of NDP52 with designated point mutations. LIR, LC3-interaction region; ZF, zinc-finger; SKICH, SKIP carboxyl homology. GFP immunoprecipitation and K63 linked polyubiquitin pull-down from RPE cells transfected with GFP alone or GFP-optineurin wild-type and mutants (**C**) or GFP-NDP52 wild-type and mutants (**D**). Western blot analysis performed with antibodies specific to indicated proteins. (**E**) Mammalian 2-hybrid assay in CHO.K1 cells expressing myosin VI tail as bait and NDP52 and optineurin wild-type and mutants as prey. Data represented as relative luciferase activity normalised to bait only control. (**F**) Mammalian 2-hybrid assay in CHOK.1 cells with TAX1BP1, NDP52, OPTN wild-type and LIR mutants as prey and myosin VI tail as bait. Data represented as relative luciferase activity normalised to bait only control.
(TIF)

S5 Fig TAX1BP1 targets to starvation induced autophagosomes with either the LC3-interaction region or the zinc-finger domains. HeLa cells were transfected with GFP-TAX1BP1 wild-type, V144S (LIR mut), Q743A/E747K/Q770A/E774K (double ZF mutant), or V144S/Q743A/E747K/Q770A/E774K (LIR and double ZF mutant), followed by amino-acid starvation for 2 hours prior to processing for confocal immunofluorescence microscopy. Cells were immunostained for GFP (green) and LC3 (red). Nuclei were labelled in blue with Hoechst. Scale bar, 20 μm.
(TIF)

S6 Fig Loss of myosin VI leads to an accumulation of ubiquitin (+) sifA mutant Salmonella, which can be rescued by re-expression of WT myosin VI. (**A**) HeLa cells were infected with sifA mutant (ΔsifA) Salmonella for 1 hour and immunostained for endogenous p62 or endogenous LC3 (green) and Salmonella (red). In addition HeLa cells were mock or myosin VI siRNA transfected, followed by infection with sifA mutant (ΔsifA) Salmonella for 4 hours. Cells were immunostained for ubiquitin (green) and Salmonella (red), nuclei labelled with Hoechst (blue), and imaged by confocal microscopy. Scale bar, 20 μm. (**B**) Representative Western blot on whole cell lysates using antibodies to myosin VI and actin. Quantitation of the % of infected cells with ubiquitin (+) ΔsifA Salmonella at 1, 4, and 8 hours (hr) post-infection. Results represent 3 independent experiments and error bars are the s.d. (**C**) HeLa parental or GFP-myosin VI WT (07 siRNA resistant) expressing cells were mock or myosin VI 07 siRNA transfected. Western blot analysis using antibodies to specified proteins indicating suppression of endogenous myosin VI expression and expression of WT rescue siRNA resistant protein. (**D**) Confocal microscopy taken from HeLa cells 4 hours post-infection with ΔsifA Salmonella (green), immunostained for ubiquitin (white), and nuclei labelled with Hoechst (blue). Scale bar, 20 μm. (**E**) Quantitation of the % of infected cells with ubiquitin (+) Salmonella 4 hours post infection in parental and WT rescue HeLa cells following myosin VI 07 siRNA transfection. Results represent 4 independent experiments and the error bars indicate the s.d.
(TIF)

S7 Fig Loss of myosin VI from the Snell’s Waltzer knockout fibroblasts leads to the accumulation of ubiquitylated sifA mutant Salmonella. (**A**) Gentamicin protection assay of wild-type (WT) and Snell’s Waltzer (SV) MEFs infected with Salmonella for indicated time points. Colonies were counted in duplicate from 5 independent experiments and results are represented as Salmonella proliferation calculated as the fold change from 2 hr. Error bars are s.d. Western blot analysis was performed on wild-type and Snell’s Waltzer whole cell lysates utilising antibodies specific to the indicated proteins. (**B**) Wild-type and Snell’s Waltzer fibroblasts were infected with ΔsifA Salmonella for the indicated time periods. The cells were processed for confocal microscopy and immunostained for ubiquitin (green) and Salmonella (red). Nuclei were labelled with Hoechst (blue). Scale bar, 20 μm. (**C**) Quantitative microscopy performed on a Cellomics VTi microscope and the area of ubiquitin fluorescence was calculated per cell in >500 cells/experiment at each given time point post Salmonella infection. Results represent 3 independent experiments and the error bars indicate the s.d.
(TIF)

S1 Table Summary of conformational constraints and statistics for the 20 accepted NMR structures of the ZF domains of human TAX1BP1. (DOC)

S2 Table Table of accession numbers for orthologues used in phylogenetic analysis in Fig 1. (XLSX)

S1 Movie Time-lapse movie illustrating recruitment of TAX1BP1 to Salmonella. RPE cells expressing GFP-TAX1BP1 were infected with cherry expressing Salmonella and imaged on a spinning disk microscope. Images were captured every 10 seconds for a period of 20 minutes. Elapsed time is displayed in min:sec.
(AVI)

S2 Movie Time-lapse movie illustrating recruitment of NDP52 to Salmonella. RPE cells expressing GFP-NDP52 were infected with cherry expressing Salmonella and imaged on a spinning disk microscope. Images were captured every 10 seconds for a period of 20 minutes. Elapsed time is displayed in min:sec.
(AVI)
