# Correction: The Autophagy Receptor TAX1BP1 and the Molecular Motor Myosin VI Are Required for Clearance of Salmonella Typhimurium by Autophagy

**DOI:** 10.1371/journal.ppat.1005433

**Published:** 2016-01-28

**Authors:** David A. Tumbarello, Paul T. Manna, Mark Allen, Mark Bycroft, Susan D. Arden, John Kendrick-Jones, Folma Buss

The order of the panel descriptions in the figure legend for Fig 5 is incorrect. Please see the complete, correct Fig 5 caption here.

**Fig 5:**
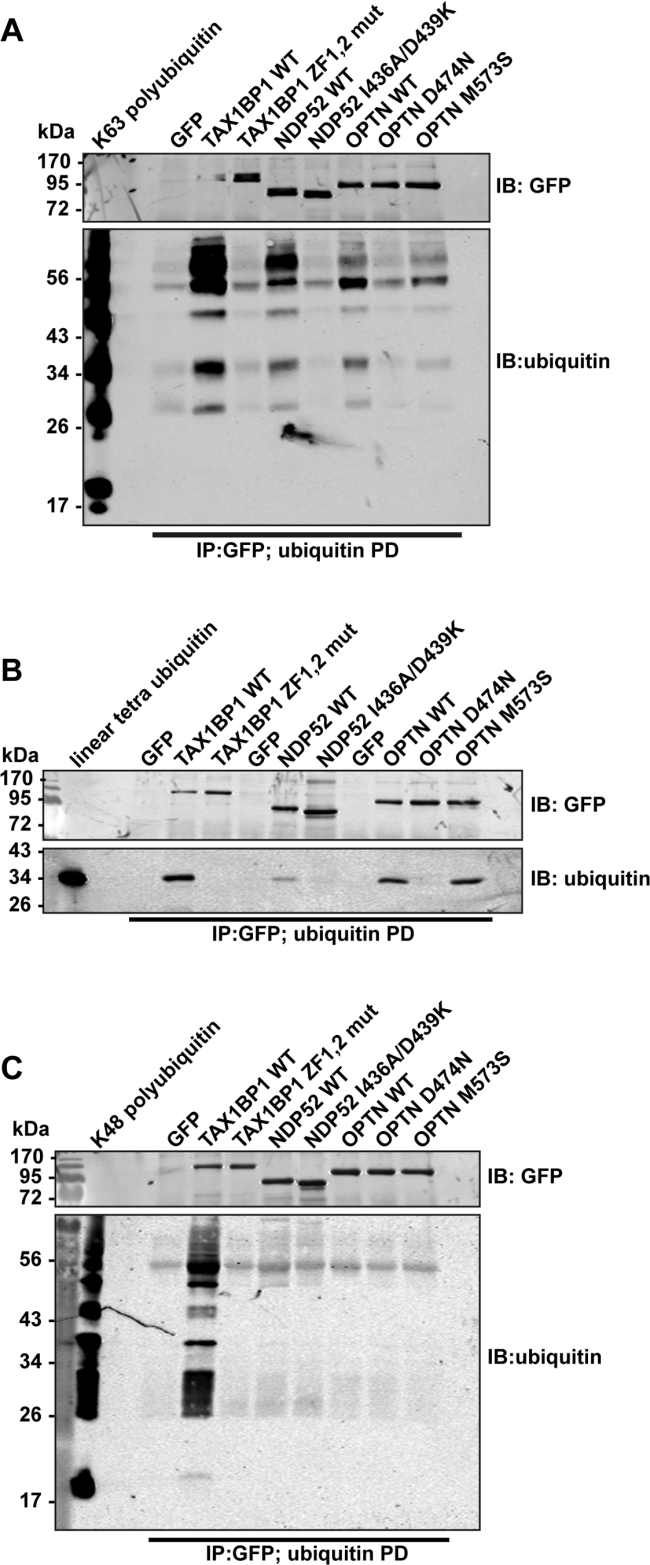
Binding of TAX1BP1, NDP52 and optineurin to different ubiquitin chain types. GFP immunoprecipitation followed by either K63-linked polyubiquitin (A), linear tetra-ubiquitin (B), or K48-linked polyubiquitin (C) pull-downs (PD) from RPE cells transfected with GFP alone, GFP-TAX1BP1, GFP-NDP52, or GFP-optineurin wild-type and mutants. Western blot analysis performed using antibodies specific for ubiquitin and GFP.
