# Supplementary figures and images for: The Autophagy Receptor TAX1BP1 and the Molecular Motor Myosin VI Are Required for Clearance of Salmonella Typhimurium by Autophagy
Source: PLoS Pathog. 2015 Oct 9;11(10):e1005174. doi: 10.1371/journal.ppat.1005174 (PMC4599966; doi:10.1371/journal.ppat.1005174)

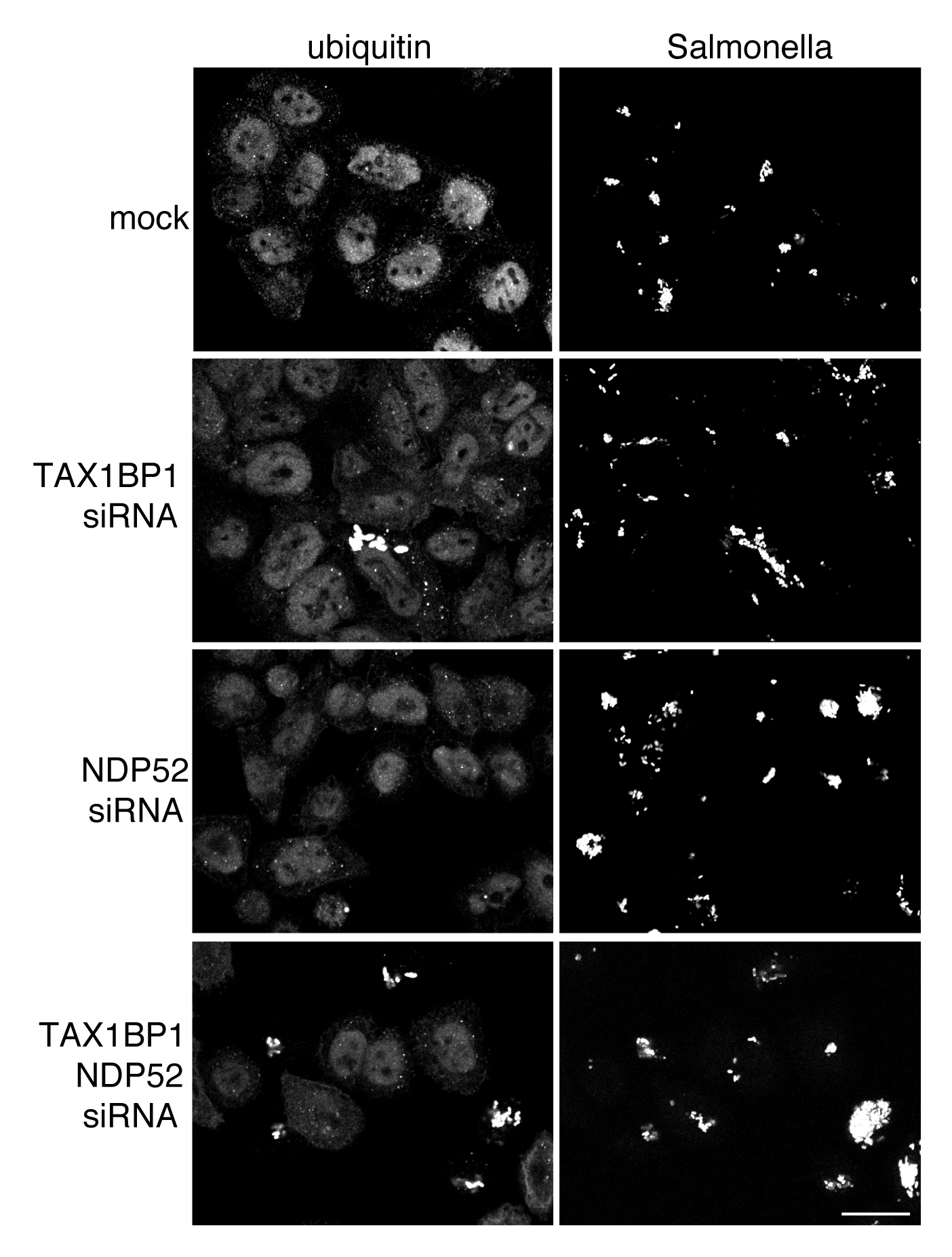

Supplement: S1 Fig — HeLa cells transfected with siRNA targeted to TAX1BP1, NDP52, or both together were subjected to an infection with mCherry expressing Salmonella for 8 hours followed by processing for confocal microscopy. Immunostaining was performed against ubiquitin. Nuclei were labelled with Hoechst (blue). Scale bar, 20 μm. (TIF) [file ppat.1005174.s001.tif]

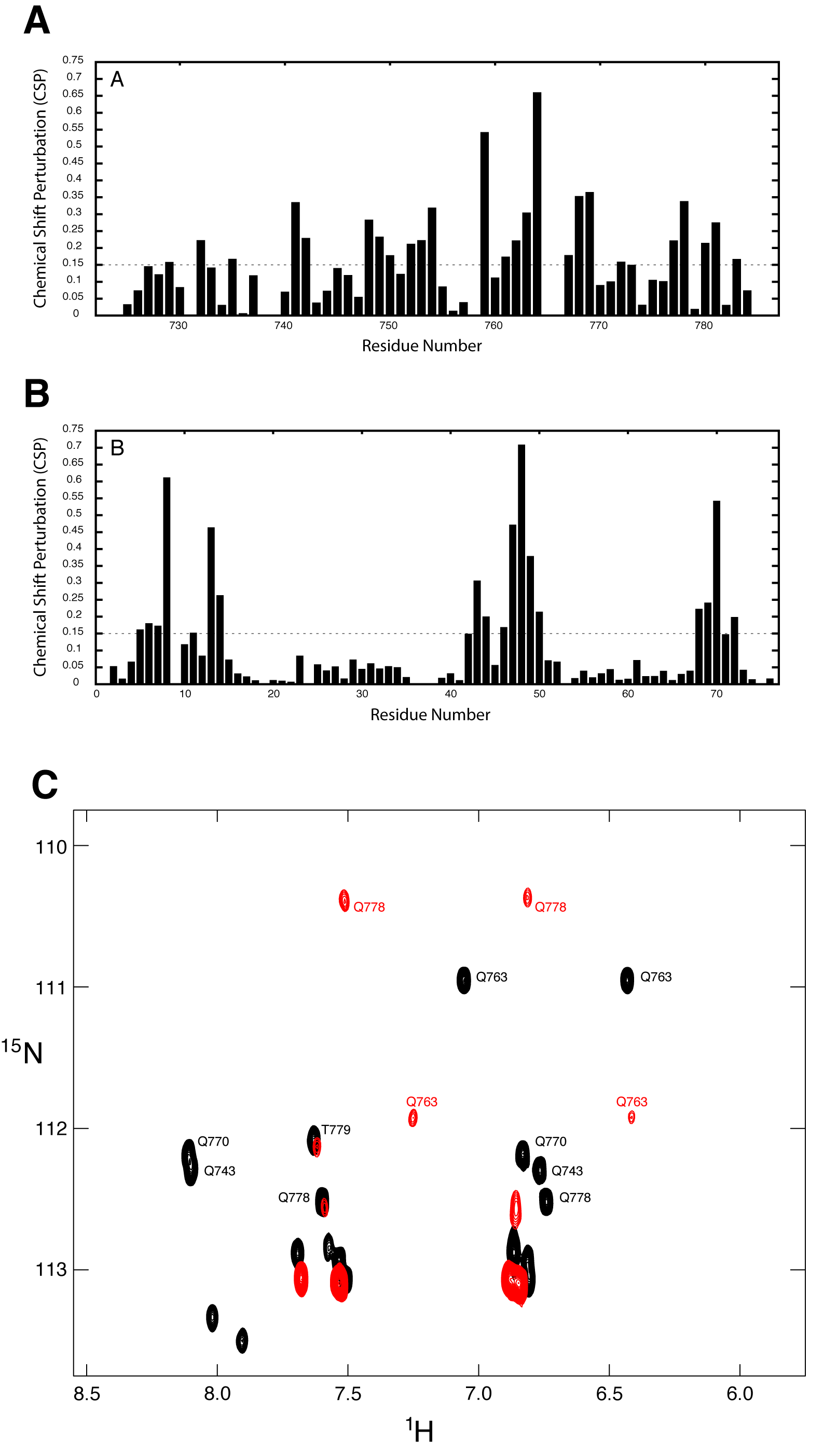

Supplement: S2 Fig — (A) Normalized cumulative chemical shift for the backbone amide groups of 15N-labeled TAX1BP1 zinc finger domains upon addition of unlabeled ubiquitin, (B) reciprocal cumulative chemical shift changes upon titration of 15N-labeled ubiquitin with unlabeled TAX1BP1 zinc finger domains, (C) section of the the1H/15N HSQC spectra of 15N labeled TAX1BP1 zinc fingers showing the resonances of the side-chain NH groups of glutamine residues in the presence and absence of unlabeled ubiquitin. The peaks that change chemical shift or decrease in intensity are labeled. (TIF) [file ppat.1005174.s002.tif]

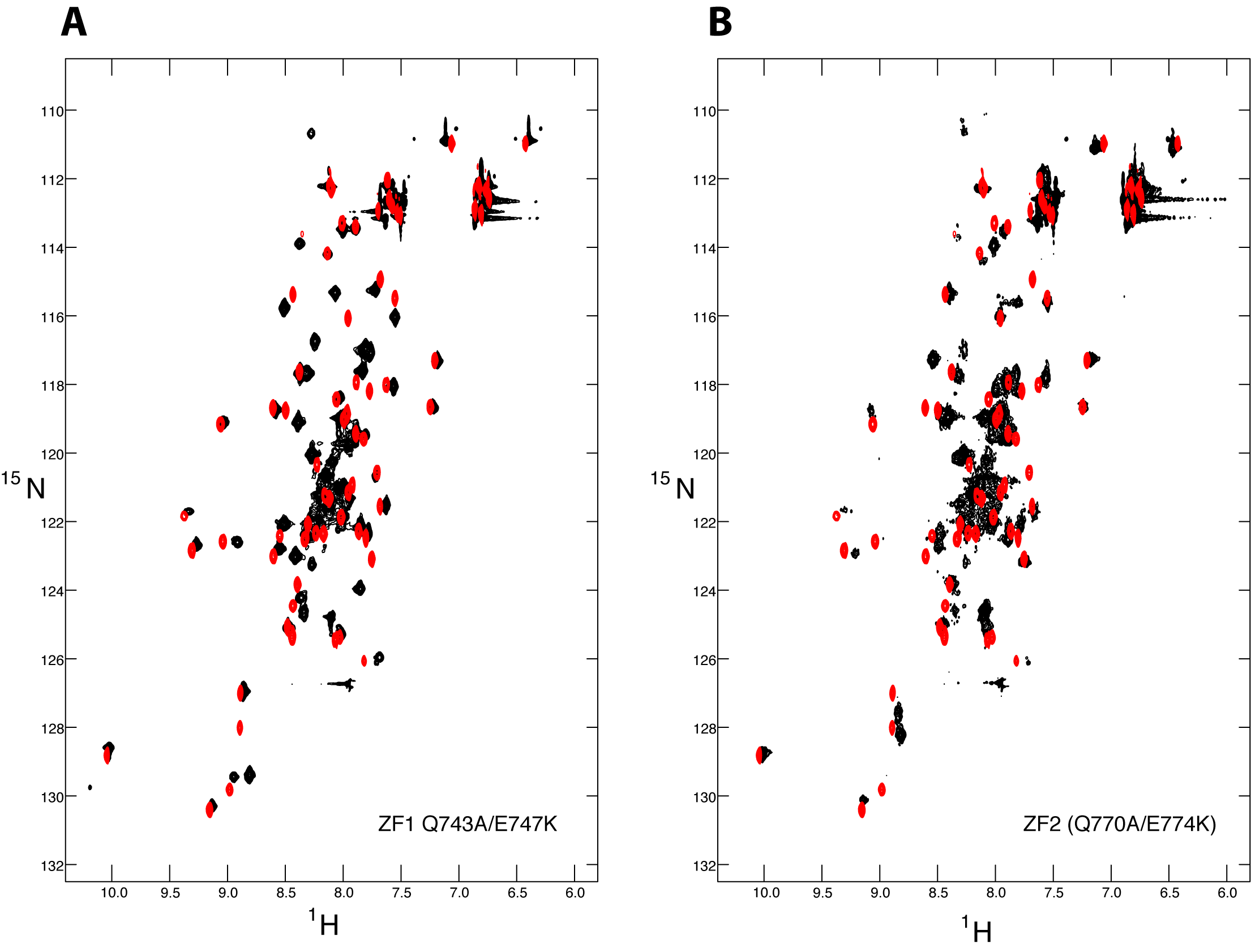

Supplement: S3 Fig — HSQC spectra of the ZF1 (A) and ZF2 (B) mutants of TAXBP1 (black) overlayed with the HSQC spectrum of wild-type TAXBP1 (red). Both of the mutant proteins are clearly folded with chemical shift in similar positions to those in the wild-type domain. (TIF) [file ppat.1005174.s003.tif]

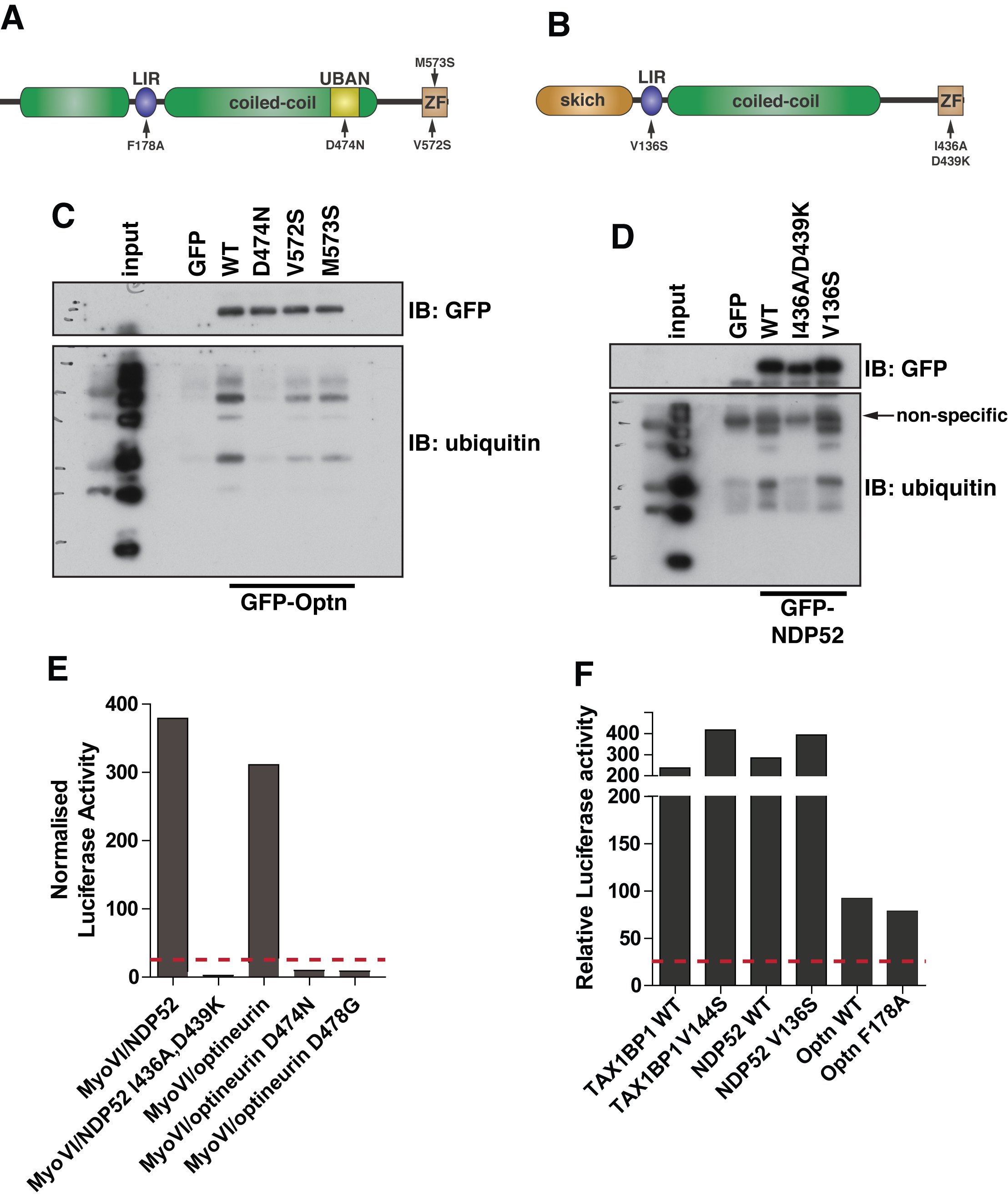

Supplement: S4 Fig — (A) Domain organisation of optineurin with designated point mutations. LIR, LC3-interaction region; UBAN, ubiquitin-binding domain in ABINs (A20-binding inhibitors of NF-κB) and NEMO; ZF, zinc-finger. (B) Domain organisation of NDP52 with designated point mutations. LIR, LC3-interaction region; ZF, zinc-finger; SKICH, SKIP carboxyl homology. GFP immunoprecipitation and K63 linked polyubiquitin pull-down from RPE cells transfected with GFP alone or GFP-optineurin wild-type and mutants (C) or GFP-NDP52 wild-type and mutants (D). Western blot analysis performed with antibodies specific to indicated proteins. (E) Mammalian 2-hybrid assay in CHO.K1 cells expressing myosin VI tail as bait and NDP52 and optineurin wild-type and mutants as prey. Data represented as relative luciferase activity normalised to bait only control. (F) Mammalian 2-hybrid assay in CHOK.1 cells with TAX1BP1, NDP52, OPTN wild-type and LIR mutants as prey and myosin VI tail as bait. Data represented as relative luciferase activity normalised to bait only control. (TIF) [file ppat.1005174.s004.tif]

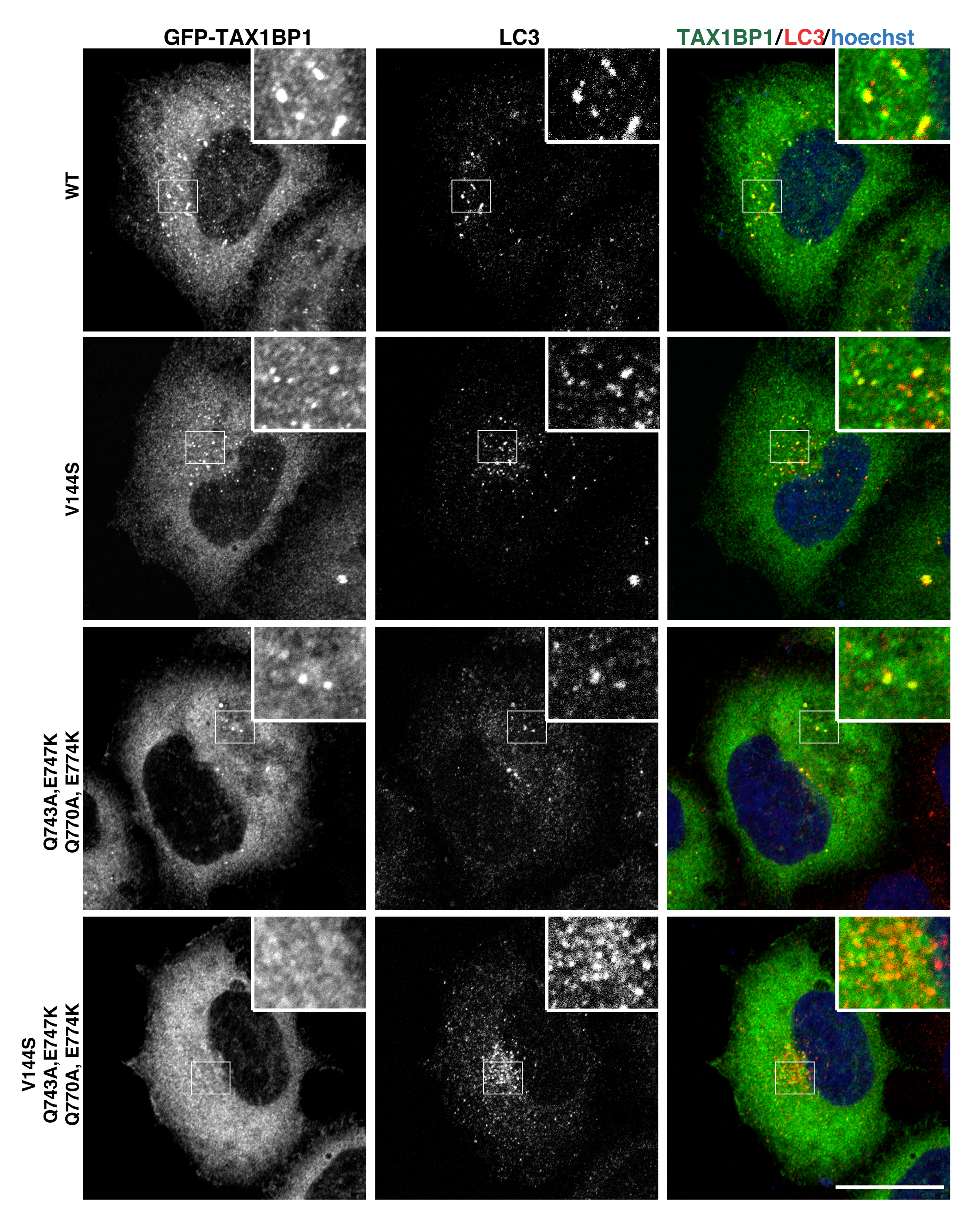

Supplement: S5 Fig — HeLa cells were transfected with GFP-TAX1BP1 wild-type, V144S (LIR mut), Q743A/E747K/Q770A/E774K (double ZF mutant), or V144S/Q743A/E747K/Q770A/E774K (LIR and double ZF mutant), followed by amino-acid starvation for 2 hours prior to processing for confocal immunofluorescence microscopy. Cells were immunostained for GFP (green) and LC3 (red). Nuclei were labelled in blue with Hoechst. Scale bar, 20 μm. (TIF) [file ppat.1005174.s005.tif]

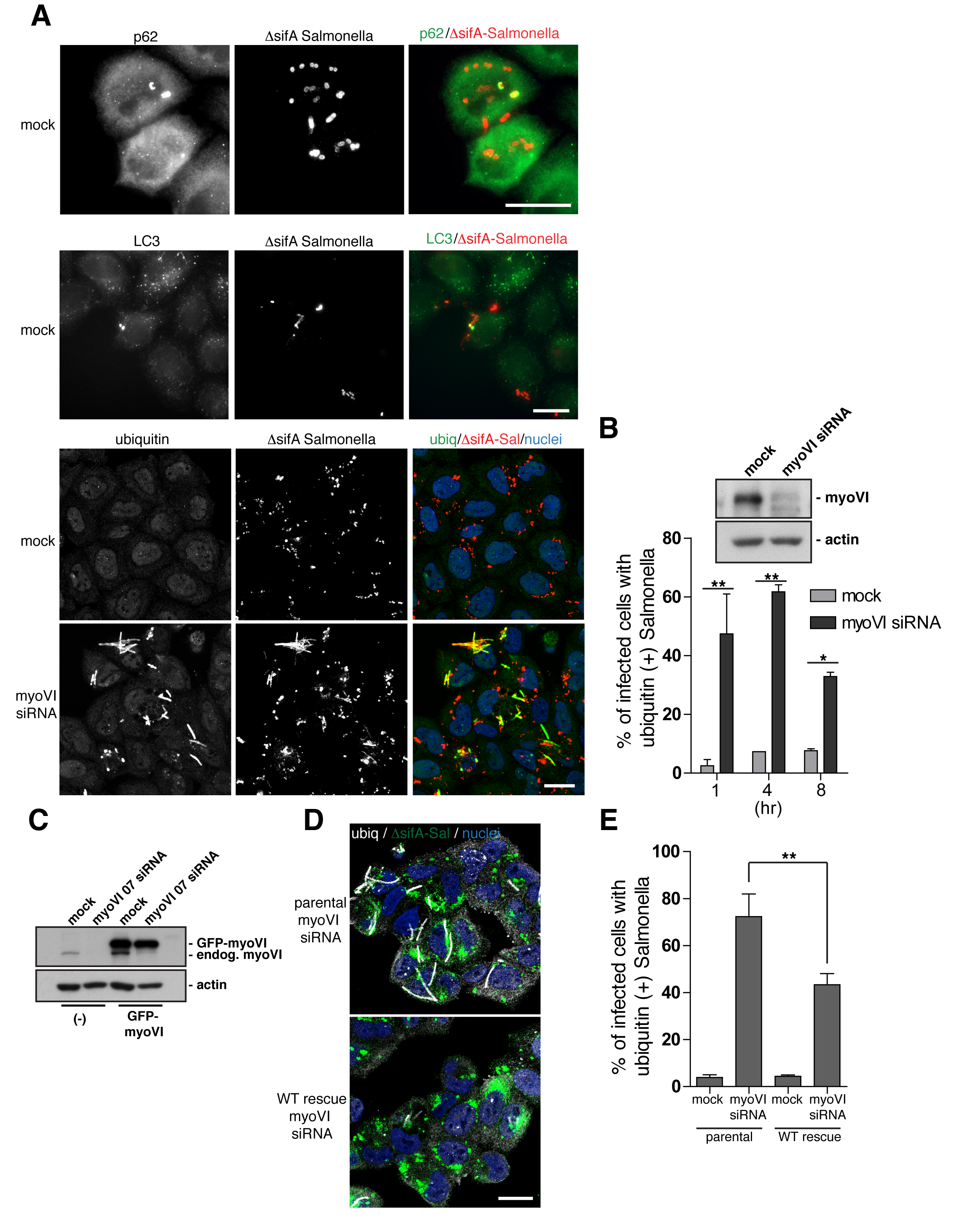

Supplement: S6 Fig — (A) HeLa cells were infected with sifA mutant (ΔsifA) Salmonella for 1 hour and immunostained for endogenous p62 or endogenous LC3 (green) and Salmonella (red). In addition HeLa cells were mock or myosin VI siRNA transfected, followed by infection with sifA mutant (ΔsifA) Salmonella for 4 hours. Cells were immunostained for ubiquitin (green) and Salmonella (red), nuclei labelled with Hoechst (blue), and imaged by confocal microscopy. Scale bar, 20 μm. (B) Representative Western blot on whole cell lysates using antibodies to myosin VI and actin. Quantitation of the % of infected cells with ubiquitin (+) ΔsifA Salmonella at 1, 4, and 8 hours (hr) post-infection. Results represent 3 independent experiments and error bars are the s.d. (C) HeLa parental or GFP-myosin VI WT (07 siRNA resistant) expressing cells were mock or myosin VI 07 siRNA transfected. Western blot analysis using antibodies to specified proteins indicating suppression of endogenous myosin VI expression and expression of WT rescue siRNA resistant protein. (D) Confocal microscopy taken from HeLa cells 4 hours post-infection with ΔsifA Salmonella (green), immunostained for ubiquitin (white), and nuclei labelled with Hoechst (blue). Scale bar, 20 μm. (E) Quantitation of the % of infected cells with ubiquitin (+) Salmonella 4 hours post infection in parental and WT rescue HeLa cells following myosin VI 07 siRNA transfection. Results represent 4 independent experiments and the error bars indicate the s.d. (TIF) [file ppat.1005174.s006.tif]

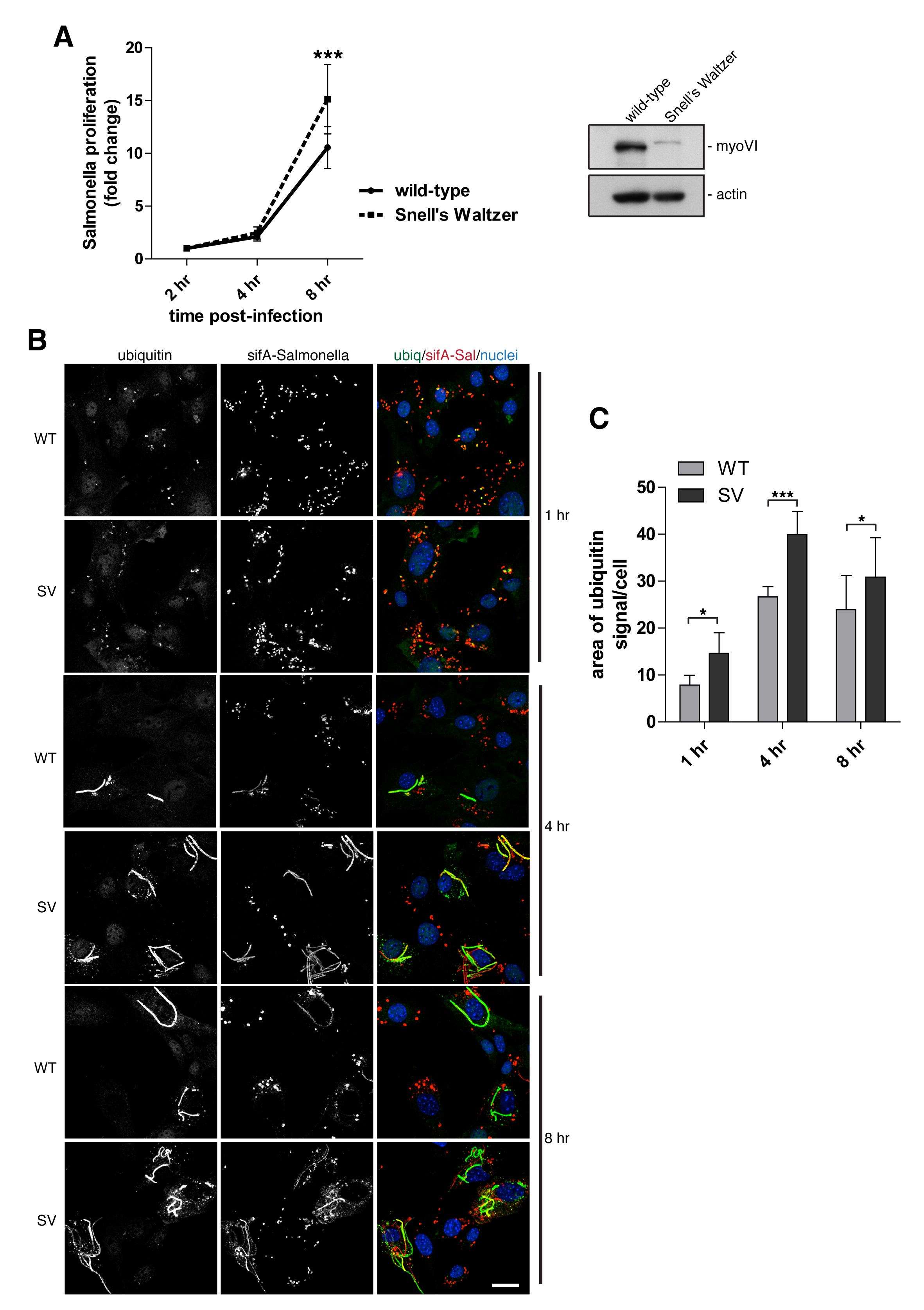

Supplement: S7 Fig — (A) Gentamicin protection assay of wild-type (WT) and Snell’s Waltzer (SV) MEFs infected with Salmonella for indicated time points. Colonies were counted in duplicate from 5 independent experiments and results are represented as Salmonella proliferation calculated as the fold change from 2 hr. Error bars are s.d. Western blot analysis was performed on wild-type and Snell’s Waltzer whole cell lysates utilising antibodies specific to the indicated proteins. (B) Wild-type and Snell’s Waltzer fibroblasts were infected with ΔsifA Salmonella for the indicated time periods. The cells were processed for confocal microscopy and immunostained for ubiquitin (green) and Salmonella (red). Nuclei were labelled with Hoechst (blue). Scale bar, 20 μm. (C) Quantitative microscopy performed on a Cellomics VTi microscope and the area of ubiquitin fluorescence was calculated per cell in >500 cells/experiment at each given time point post Salmonella infection. Results represent 3 independent experiments and the error bars indicate the s.d. (TIF) [file ppat.1005174.s007.tif]
