# Supplementary material for: The Autophagy Receptor TAX1BP1 and the Molecular Motor Myosin VI Are Required for Clearance of Salmonella Typhimurium by Autophagy
Source: PLoS Pathog. 2015 Oct 9;11(10):e1005174. doi: 10.1371/journal.ppat.1005174 (PMC4599966; doi:10.1371/journal.ppat.1005174)
Supplement: S1 Table — (DOC) [file ppat.1005174.s008.doc]

|  | |
| --- | --- |
| **Summary of conformational constraints and statistics for the 20 accepted NMR structures of the ZF domains of human TAX1BP1** | |
| **Structural constraints** | |
| Intra-residue  Sequential  Medium-range ( 2 ≤ |i-j| ≤ 4 )  Long-range ( |i-j| > 4 )  Dihedral angle constraints  TALOS constraints  Distance constraints for 15 hydrogen bonds  Zinc co-ordination constrainsts  Total | 485  348  332  408  25  98  30  20  1746 |
| **Statistics for accepted structures** | |
|  | |
| Statistical parameters (±SD) | |
| Rms deviation for distance constraints  Rms deviation for dihedral constraints | 0.0069Å ± 0.0003Å  0.343^o^ ± 0.050^o^ |
| Mean CNS energy term (kcal mol^-1^ ±SD) | |
| E (overall)  E (van der Waals)  E (distance constraints)  E (dihedral and TALOS constraints) | 66.18 ± 2.13  19.49 ± 1.30  5.75 ± 0.46  1.80 ± 0.43 |
| Rms deviations from the ideal geometry (±SD) | |
| Bond lengths  Bond angles  Improper angles | 0.0014 Å ± 0.0001Å  0.344 ^o^ ± 0.0035 ^o^  0.197 ^o^ ± 0.008 ^o^ |
| Average atomic rmsd from the mean structure (±SD) | |
| Residues 726-782 (N, Cα, C atoms)  Residues 726-782 (all heavy atoms) | 0.367 Å ± 0.141 Å  0.781 Å ± 0.115 Å |
